# Immunohistochemistry in intrahepatic cholangiocarcinoma: histological subtyping and drug selection

**DOI:** 10.3389/fonc.2025.1653534

**Published:** 2025-10-06

**Authors:** Han Wang, Miao-Xia He, Wen-Ming Cong, Hui Dong

**Affiliations:** ^1^ Department of Pathology, Eastern Hepatobiliary Surgery Hospital, Naval Medical University, Shanghai, China; ^2^ Department of Pathology, Changhai Hospital, Naval Medical University, Shanghai, China

**Keywords:** large duct type, small duct type, cholangiolocarcinoma, iCCA with ductal plate malformation pattern, target therapy, immunotherapy, molecular pathology, IHC

## Abstract

**Introduction:**

Since the release of the *World Health Organization* (*WHO*) *Classification of Tumours-Digestive System Tumours* in 2019, the pathology of intrahepatic cholangiocarcinoma (iCCA) has entered an era of integrated diagnosis, encompassing gross classification, histological subtyping, as well as drug molecular target screening. Substantial evidence indicates that the histological subtypes of iCCA are significantly associated with the detection frequency of molecular targets relevant to the targeted therapy and immunotherapy. Through rational immunohistochemistry profiling, patients with iCCA can be precisely diagnosed and individually managed.

**Methods:**

A thorough literature search was conducted using terms pertinent to the pathological diagnosis, histological subtyping, targeted therapy, and immunotherapy of iCCA. The content related to immunohistochemistry was summarized.

**Results:**

In the first part, we summarize the immunohistochemical markers for the histological subtype of iCCA (e.g., large duct type iCCA, small duct type iCCA), with a particular emphasis on their percentage of positive cases, expression location, and association with prognosis. Subsequently, a summary of the immunohistochemical markers for targeted therapy and immunotherapy of iCCA is performed, focusing on the consistency between immunohistochemistry and molecular detection, optimal clone, and prognostic significance.

**Conclusions:**

This review summarizes the critical role of immunohistochemistry in the pathological diagnosis of iCCA. It is noted that any diagnosis must be made by integrating comprehensive information. A pathological diagnosis merely based on immunohistochemical results is unreasonable. The development of subtype-specific and drug-targeted antibodies holds promise for refining iCCA precise diagnosis and therapeutic stratification.

## 1 Introduction

Intrahepatic cholangiocarcinoma (iCCA) is a malignant intrahepatic epithelial neoplasm with biliary differentiation, which may originate from the lining epithelium of the intrahepatic bile duct, peritubular gland, and liver progenitor cell, accounting for approximately 10%~15% of primary liver cancers (1, 2). The 5th edition of the *World Health Organization* (*WHO*) *Classification of Tumours-Digestive System Tumours* recommends classifying iCCA into large duct type, small duct type and other less common histological subtypes (2). Histological subtyping has become an essential component of the standardized diagnosis of iCCA, and the use of appropriate immunohistochemical markers significantly enhances classification accuracy, particularly in tumors with overlapping or heterogeneous pathologic features (3).

With the success of clinical trials involving small-molecule inhibitors targeting *fibroblast growth factor receptor* (*FGFR*) *2* rearrangements (4), *isocitrate dehydrogenase* (*IDH*) *1* mutations (5) and programmed cell death ligand 1 (PD-L1) inhibitors (6, 7), it is now recommended that patients with advanced-stage iCCA undergo precise drug-related profiling to develop personalized treatments (8). Many studies have indicated that there are considerable differences in molecular alterations between large and small duct type iCCA, which facilitates the integration of histological subtyping with pharmacological options (9). Most molecular targets are recommended to be identified through next-generation sequencing (NGS). Considering the high cost and relatively complex procedures of NGS detection and analysis, several studies have attempted to use immunohistochemistry to screen suitable candidates for target therapy and immunotherapy.

Focusing on these concerns, we aim to summarize existing studies about the immunohistochemical practice for histological subtyping and drug selection in iCCA, providing a practical reference for pathologists and clinicians.

## 2 Histological subtypes of iCCA

The histological subtype of iCCA was first characterized in the 20th century. In 1977, Okuda et al. categorized iCCA into ‘‘hilar type’’ and ‘‘peripheral type’’ based on autopsy findings, proposing that the former resembled extrahepatic cholangiocarcinoma, while the latter shared similarities with hepatocellular carcinoma (10). Subsequent research examined the biological behavior and prognosis of iCCA within this classification and validated its rationale (11, 12). However, as hilar cholangiocarcinoma was a distinct tumor entity, this classification required a more suitable term, Nakanuma Y. et al. introduced the classification of ‘‘large duct type’’ and ‘‘small duct type’’ corresponding to ‘‘hilar type’’ and ‘‘peripheral type’’ in 2010 (13). This classification was incorporated into the 5th edition of the *WHO Classification of Tumours-Digestive System Tumours* (2). Currently, multiple guidelines and consensuses recommend this histological concept of iCCA (1, 8). Although most subtypes of iCCA can be identified through morphological assessment, the application of immunohistochemical markers is vital for subtype clarification and regional delineation when morphological characteristics are ambiguous, overlapping, or coexistent (3, 14). We aim to review the percentage of positive cases, localizations of expression, and prognostic implications of these markers. It should be noted that although some studies did not use the terms ‘‘large duct type’’ and ‘‘small duct type’’, the descriptions of the classification criteria in these studies were consistent with the corresponding concepts. we have also included these studies in our review (**Table 1**).

**Table 1:** Subtyping diagnostic immunohistochemical markers for iCCA.

Subtypes	Immunohistochemical markers
Large duct type iCCA	S100P, MUC5AC, MUC6, TFF1, AGR2, MMP7, pCEA, CA19-9, CLDN18, CLDN18.2, Smad4/DPC4, EVI1, PPP1R1B
Small duct type iCCA	CRP, N-cadherin, CD56, Nestin, OPN, TUBB3, FGB
Cholangiolocarcinoma	CD56, MUC1, MECA-79, Nestin, MLH1, PMS2, MSH2, MSH6
iCCA with DPM pattern	CD56, MUC1, ARID1A
Adenosquamous carcinoma/squamous cell carcinoma	p40, p63
Sarcomatoid carcinoma	Vimentin, INI1, BRG1
Cholangioblastic iCCA	α-inhibin
Enteroblastic iCCA	SALL4

### 2.1 Large duct type iCCA

Large duct type iCCA generally manifests as periductal infiltrating type or periductal infiltrating type + mass-forming type grossly and is histologically characterized by a ductal or tubular pattern with columnar to cuboidal epithelium rich in mucus. The WHO Classification lists the following markers for large duct type iCCA: S100 calcium-binding protein P (S100P), mucin (MUC) 5AC, MUC6, trefoil factor 1 (TFF1), anterior gradient protein (AGR) 2, and matrix metalloproteinase 7 (MMP7).

S100P, a member of the S100 family containing 95 amino acids, was initially identified in the placenta. It is associated with the growth, invasion, and metastasis of various tumors (15). S100P is the most widely recognized marker for large duct type iCCA. The percentage of positive cases of S100P in large duct type iCCA was reported to range from 68.5% to 100.0%, whereas in small duct type iCCA, it ranged from 0.0% to 57.6%, demonstrating a significant difference (16–28). Another study reported a percentage of positive cases of only 9.1% (1/11) for S100P in large duct type iCCA (29), possibly due to small sample size, antigen degradation during prolonged archival of tissue (some blocks archived >10 years), limited tumor area in tissue microarrays, and stringent evaluation criteria (>20% strong tumor cell labeling). Different clones of S100P antibody showed varying sensitivity and specificity (22). S100P typically shows cytoplasmic and/or nuclear expression in tumor cells, with nuclear staining being more diagnostically significant. Notably, neutrophils exhibit positive S100P staining, which may cause potential misinterpretation (12). Many studies concluded that S100P expression was significantly associated with poor prognosis in iCCA (12, 30), suggesting that large duct type iCCA has a worse survival compared to small duct type iCCA (18).

As a member of the MUC family characterized by foveolar-type mucin, MUC5AC demonstrates superior diagnostic value in distinguishing large duct type iCCA from small duct type iCCA. The reported percentage of positive cases of MUC5AC in large duct type iCCA ranges from 50.0% to 96.7%, whereas in small duct type iCCA, this percentage was significantly lower, at 1.8% to 25.0% (23, 25, 31–34). MUC5AC expression localizes in the cytoplasm of tumor cells, sometimes restricted to the apical cytoplasm, and is also observed in the mucus secreted into the glandular lumen. While rare small duct type iCCAs show focal expression of MUC5AC, such expression is strictly limited to apical cytoplasm of tumor cells and mucus within the glandular lumen. Positive expression of MUC5AC is associated with poor prognosis in iCCA (35), which is similar to S100P (18).

MUC6 is another representative of the MUC family of gastric pyloric gland-type mucin. Large duct type iCCA (13.7%~33.3%) exhibited a marginal but not significant higher percentage of positive cases of MUC6 expression than that of small duct type iCCA (3.8%~20.0%) (25, 31, 33, 34). The localization of MUC6 expression parallels that of MUC5AC. In terms of prognosis, MUC6 has an opposite trend to MUC5AC, with its high expression being positively correlated with a favorable prognosis in patients with iCCA (31, 36–38). However, no studies have elucidated the underlying mechanisms responsible for this correlation.

TFF1 plays a crucial role in the mucosal defense and repair of the gastrointestinal tract, being secreted with mucus onto the epithelial surface (39). TFF1 predominantly localizes in large bile ducts and diseased liver tissues, probably representing a feedback response to biliary injury (40). The percentage of positive cases of TFF1 in large duct type iCCA ranges from 54.9% to 91.8%, while in small duct type iCCA, it ranges from 12.0% to 48.1% (17, 19, 28, 32, 41). TFF1 staining is confined to the cytoplasm of tumor cells and the luminal surface of tumor glands (42). Some scholars have found that high expression of TFF1 indicates more invasive and metastatic potential in patients with liver fluke-related cholangiocarcinoma, which mainly were large duct type iCCA (32, 43).

The AGR family consists of three members: AGR1, AGR2, and AGR3, which are involved in the regulation of the redox reactions. AGR2 is an atypical member of this family, and its physiological function remains poorly understood. However, AGR2 is connected with the tumor progression of various cancers (44, 45). AGR2 was initially found positive expression in fibrolamellar hepatocellular carcinoma, while classic hepatocellular carcinoma and hepatocellular adenoma exhibited negative expression (46). Subsequent studies found that AGR2 was expressed in the columnar epithelial cell covering the intrahepatic and hilum large bile duct, as well as the peribiliary gland, gallbladder epithelial cell, and large duct type iCCA (47). Two studies reported that the percentage of positive cases of AGR2 in large duct type iCCA varied between 58.9% and 86.7%, while in small duct type iCCA, it varied between 19.5% and 33.3% (17, 41). AGR2 staining is concentrated in the cytoplasm of tumor cells. To date, no studies have investigated the prognostic significance of AGR2 in iCCA.

MMP7 is one of the gene products of the *MMP* family, involved in the proteolytic processing of extracellular matrix components, including collagen, proteoglycans, laminin, and fibronectin. MMP7 overexpression in iCCA was demonstrated to be strongly associated with poor outcomes (48, 49). Itatsu K et al. further demonstrated the significance of MMP7 in large duct type iCCA, with a percentage of positive cases of 91.2% (31/34), while noting absent expression in normal bile ducts (50). However, data on the positivity percentage of MMP7 in small duct type iCCA patient remain limited. MMP7 expression is circumscribed in the cytoplasm of tumor cells.

Additionally, some scholars have suggested other potential immunohistochemical markers of large duct type iCCA, including polyclonal carcinoembryonic antigen (pCEA), carbohydrate antigen 19-9 (CA19-9), claudin (CLDN) 18/CLDN18.2, SMAD family member 4 (Smad4/DPC4), ecotropic virus integration site 1 protein homolog (EVI1), and protein phosphatase 1 regulatory inhibitor subunit 1B (PPP1R1B).

CEA is among the most frequently utilized serum tumor markers in clinical practice. Antibodies against CEA have been employed in the histopathological diagnosis of various tumors (51). Notably, pCEA has been found to be useful in liver tumors. A recent study indicated that pCEA can effectively differentiate between large duct type (12/18, 66.7%) and small duct type iCCA (1/11, 9.1%), with a significantly higher percentage of positive cases observed in the former. The staining pattern for pCEA is characterized by diffuse cell membrane expression (52). To date, no studies have been published investigating the relationship between its expression and the prognosis of iCCA.

CA19–9 is another common serum tumor marker, frequently positive in pancreatic cancer, cholangiocarcinoma, and gastric cancer (53). Most immunohistochemical studies on CA19–9 have focused on distinguishing iCCA from hepatocellular carcinoma. The percentage of positive cases of CA19–9 in iCCA ranges from 45.5% to 100.0%, which is significantly higher than that observed in hepatocellular carcinoma (54–58). Studies demonstrated that staining of CA19–9 could aid in differentiating subtypes of iCCA, with a percentage of positive cases of 72.7% (8/14) in large duct type iCCA and 8.4% (13/155) in small duct type iCCA (29); the corresponding percentages of strongly positive expression were 50.0% (50/100) and 35.0% (35/100), respectively (59). CA19–9 positive staining is shown in the cytoplasm of tumor cells. No study has focused on the relationship between CA19–9 expression and the prognosis of iCCA.

CLDN18 was initially identified as a new downstream target gene of the thyroid-specific enhancer-binding protein/NK2 homeobox 1 homologous domain transcription factor. CLDN18 is spliced into two specific isoforms expressed in lung and gastric tissues, with selective splicing of exons 1a and 1b forming CLDN18.1 and CLDN18.2, respectively. CLDN18.1 is mainly expressed in normal lung tissue and lung cancer cells, while CLDN18.2 is restricted to gastric tissue under normal conditions and appears on the cell membrane in cancerous tissues, including iCCA (60, 61). Tanaka M et al. demonstrated that the percentage of positive cases of CLDN18 was higher in large duct type iCCA (69.0%, 29/42) compared with that in small duct type iCCA (12.7%, 7/55) (34). CLDN18.2 was also reported to have value in distinguishing subtypes of iCCA, with a percentage of positive cases of 41.2% (42/102) and 10.0% (2/20) in large and small duct type iCCA, respectively (19). Their positive staining localizes to the cell membrane of tumor cells. Although their prognostic and treatment significance in iCCA remains unconfirmed, Zolbetuximab, a monoclonal antibody targeting CLDN18.2, has shown efficacy in patients with CLDN18.2-positive gastric or gastroesophageal junction adenocarcinoma (62). Potential studies in associated iCCA patients are well worth exploring.

Smad4/DPC4 belongs to a family of signal transduction proteins that are phosphorylated and activated by transmembrane serine/threonine receptor kinases in response to transforming growth factor beta signaling through multiple pathways. This gene functions as a tumor suppressor, and the inactivation of Smad4/DPC4 is most prominently observed in pancreatic cancer (63). In iCCA, loss of expression of Smad4/DPC4 occurs more frequently in large duct type iCCA patients than in small duct type iCCA patients (33.3%, 7/21 vs. 3.8%, 1/26) (33), and is considerably associated with a poorer histological grade, advanced clinical stage, as well as metastasis (64, 65).

EVI1, a recognized oncogenic transcription factor in hematopoietic cells, enhances the oncogenic potential of pancreatic cancer by upregulating *Kirsten ratsarcoma viral oncogene homolog* (*KRAS*) expression. According to a study by Tanaka M et al., large duct type iCCA exhibited a higher percentage of positive cases of EVI1 positivity (33 out of 42, 78.6%) compared to small duct type iCCA (18 out of 55, 32.7%). In iCCA, EVI1 is primarily expressed in the nuclei of tumor cells. In contrast, non-neoplastic intrahepatic biliary epithelium shows either no or minimal EVI1 expression. Hepatocytes show no or only very weak EVI1 positivity. Vascular endothelial cells do not show any immunoreactivity for EVI1. Patients with EVI1-positive iCCA had significantly poorer overall survival and recurrence-free survival rates (34).

PPP1R1B inhibits protein phosphatase-1 and protein kinase A, with demonstrated prognostic relevance in breast cancer and pancreatic cancer (66, 67). Recently, PPP1R1B was identified as a potential immunohistochemical marker for histosubtyping of iCCA, with a percentage of positive cases of 90.9% (40/44) in large duct type and 4.0% (2/50) in small duct type iCCA. PPP1R1B is expressed in the common bile duct but not in intrahepatic bile duct. The positive staining of PPP1R1B localizes in the cytoplasm of tumor cells. The expression of PPP1R1B promoted cell proliferation, migration, and invasion and indicated a worse prognosis of iCCA (28).

**Figure 1:**
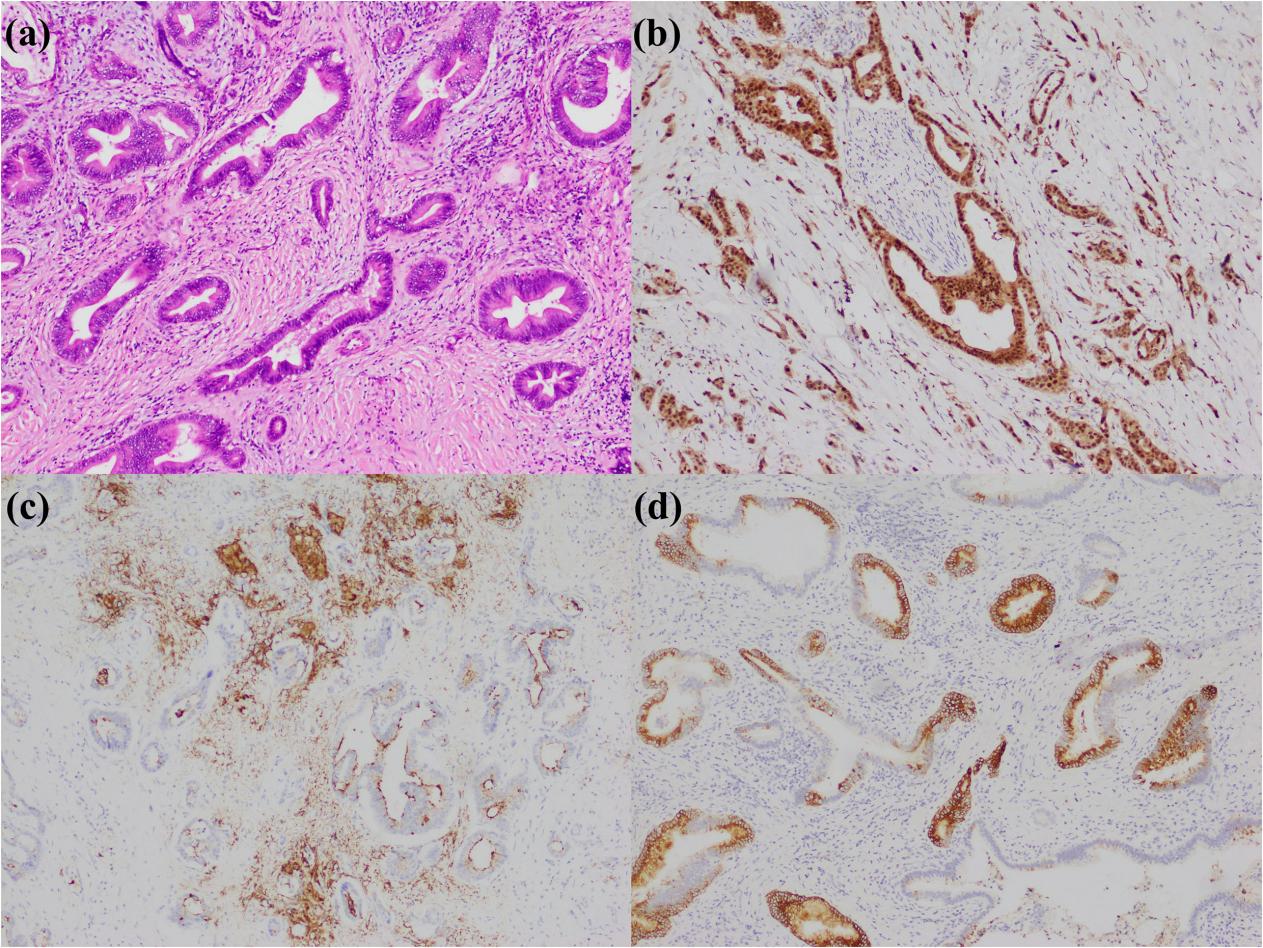
Large duct type iCCA. **(a)** histological morphology; **(b)** S100P positive; **(c)** MUC5AC positive; **(d)** MUC6 positive (magnification: 100×).

In summary, the immunohistochemical markers of large duct type iCCA are frequently expressed in the cytoplasm of tumor cells and are closely associated with secreted mucus (**Figure 1**). Consequently, numerous studies utilized Alcian Blue staining or Alcian Blue-Periodic Acid-Schiff staining to differentiate subtypes of iCCA. Positive staining is indicative of large duct type iCCA (percentage of positive cases of mucus staining in large duct type iCCA vs. small duct type iCCA: 82.0%~100% vs. 10.7%~45%) (18, 19, 21, 23, 52, 59, 68). However, since adenocarcinomas from other organs can produce similar mucus and metastasize to the liver, these markers are primarily used to distinguish large duct type iCCA from small duct type iCCA and should be cautiously employed when differentiating large duct type iCCA from other liver-metastatic adenocarcinomas. Organ-specific markers for large duct type iCCA remain undefined. Due to the same embryonic origins, adenocarcinomas arising from the pancreas, ampulla, stomach, extrahepatic bile ducts, and gallbladder exhibit highly similar immunohistochemical profiles to large duct type iCCA. In such cases, histological evidence of premalignant lesions is essential, such as biliary intraepithelial neoplasia or intraductal papillary neoplasms of the bile ducts. Furthermore, large duct type iCCA typically demonstrates abundant fibrous stroma and dilated mucin-rich tumor glands compared to metastatic adenocarcinomas to the liver, though this is not absolute. Additionally, imaging findings and clinical information should be emphasized. Metastatic adenocarcinomas often manifest as multiple subcapsular lesions accompanied by tumors in other organs and corresponding clinical symptoms, whereas large duct type iCCA predominantly shows infiltrative growth along the intrahepatic bile ducts near the porta hepatis. When the diagnosis is uncertain despite these considerations, pathological reports should employ cautious terminology and recommend multidisciplinary treatment. Given the significant differences in treatment, organ-specific immunohistochemical markers for adenocarcinomas originating from the upper gastrointestinal tract, large bile ducts, and pancreas are critically needed.

### 2.2 Small duct type iCCA

Small duct type iCCA typically shows as a mass-forming type and is morphologically characterized by a tubular pattern with low columnar to cuboidal cells lacking mucus. According to the 5th edition of the WHO Classification, immunohistochemical markers for small duct type iCCA include C-reactive protein (CRP), N-cadherin, and CD56.

CRP is an acute inflammatory protein primarily produced by the liver (69). It is one of the most sensitive and specific markers for small duct type iCCA with its percentage of positive cases in small duct type iCCA ranging from 65.0% to 100.0%, while in large duct type iCCA it ranges from 5.3% to 71.7% (19, 22, 25, 52, 59, 70). Furthermore, two studies found that *CRP* was the most markedly differentially expressed gene between small and large duct type iCCA (19, 71). Positive staining of CRP is observed in the cytoplasm of tumor cells. Notably, as CRP is a secreted protein, it often appears as diffuse strong positive staining in tumor stroma in cases with pronounced inflammation, which can affect the interpretation of tumor cell staining intensity and extent. Moreover, CRP may present simple membrane staining in tumor cells, which has no clear diagnostic significance for small duct type iCCA. Additionally, granular cytoplasmic staining of CRP in the mucosal epithelium of intrahepatic large bile ducts and the periductal glands intermixed within the lesions of large duct type iCCA may result in misdiagnosis. These three scenarios may be potential reasons for the higher percentage of positive cases of CRP observed in large duct type iCCA in some studies (59, 70). Therefore, it is essential to clarify the expression position of CRP to make an accurate diagnosis. A high expression of CRP in iCCA indicated a favorable prognosis (72), suggesting that the prognosis of small duct type iCCA is better than that of large duct type iCCA (19, 25, 68).

N-cadherin, the expression product of *cadherin 2* (*CDH2*), is associated with epithelial-mesenchymal transition and mediates tumor cell migration (73). It is another one of the most classic markers for small duct type iCCA. The noted percentage of positive cases of N-cadherin in small duct type iCCA varied between 55.0% and 95.5%, whereas in large duct type iCCA, it varied between 5.6% and 49.1% (17–19, 22, 23, 25, 28, 41, 52, 59, 74). N-cadherin staining appears on the membrane of tumor cells. Although the sensitivity and specificity of N-cadherin for small duct type iCCA may be slightly lower than those of CRP, N-cadherin does not manifest strong background staining, making it easier to interpret than CRP and suitable for use in conjunction with CRP. The impact of N-cadherin on the prognosis of iCCA remains controversial. A study by a Thai team suggested that high expression of N-cadherin was indicative of a poor prognosis for iCCA (75), while a study by a Taiwanese team suggested that N-cadherin expression did not represent a different prognosis (72). These discrepancies may be attributed to the different etiological factors and proportions of histological subtypes in the studied populations.

CD56 serves as a marker of liver progenitor cells (76) and is expressed in canals of Hering, bile ductules, and interlobular bile ducts, as well as ductular reactions, bile duct adenomas, and biliary hamartomas (77–79). Consequently, it is recognized as an immunohistochemical marker for small duct type iCCA, particularly its specific subtypes: cholangiolocarcinoma and iCCA with ductal plate malformation (DPM) pattern (80). The percentage of positive cases of CD56 in small duct type iCCA (16.2% to 82.1%) was higher than that in large duct type iCCA (0.0% to 25.5%) (18, 20, 22, 23, 25, 28, 29, 59). CD56 staining is localized in the cell membrane and cytoplasm of tumor cells and is frequently focally positive in small duct type iCCA. Its positivity correlates with the morphological features of cholangiolocarcinoma, although this association is not absolute. To date, there have been no studies focusing on the prognostic significance of CD56 expression in iCCA. Given that CD56 is often expressed in cholangiolocarcinoma, which generally has a better prognosis (20, 81–83), it is reasonable to infer that patients with CD56 expression in iCCA have a more favorable prognosis.

In addition to the aforementioned immunohistochemical markers, several other valuable markers for small duct type iCCA were identified, including nestin, osteopontin (OPN), class III β-tubulin (TUBB3), and fibrinopeptide B (FGB).

Nestin is commonly utilized for the diagnosis of nervous system disorders. Subsequently, a multicenter study discovered its specificity in combined hepatocellular-cholangiocarcinoma and its association with poor prognosis of this tumor entity. This study also involved 204 iCCAs and found that Nestin staining was positive in 120 patients (positive percentage: 58.8%) (84). Consequently, Sasaki M et al. published two papers discussing the diagnostic significance of Nestin in iCCA, reporting that percentage of positive cases ranged from 40.9% to 47.2% in small duct type iCCA and 3.3%~5.0% in large duct type iCCA (85, 86), suggesting its potential as a marker for differentiating subtypes of iCCA. However, some scholars argued that Nestin-positive tumors should be directly classified as cholangiolocarcinoma, whereas classic small duct type iCCA did not express Nestin (80). Nestin-positive staining localizes in the cytoplasm of tumor cells and is not expressed in the non-tumorous small bile duct, large bile duct, or hepatocyte, while the vessel of liver show Nestin-positive expression. It also should be noted that given the comparable expression status of Nestin observed in combined hepatocellular-cholangiocarcinoma and iCCA (84), careful consideration should be given to the possibility of the intermediate cell carcinoma component within combined hepatocellular-cholangiocarcinoma when diagnosing small duct type iCCA (87). This is crucial since morphological differentiation can be challenging between intermediate cell carcinoma and small duct type iCCA. Additionally, the potential diagnosis of combined hepatocellular-cholangiocarcinoma should be considered when encountering small duct type iCCA lacking expression of Nestin in needle biopsy specimens (87).

OPN is a multifunctional protein that plays important roles in various conditions, including cardiovascular diseases, cancers, diabetes, and kidney stones, and in the processes of inflammation, biomineralization, cell viability, and wound healing (88). Song G et al. conducted single-cell sequencing for 14 patients with iCCA, identifying two molecular subtypes: *S100P* ^-^ *secreted phosphoprotein 1* (*SPP1*)^+^ iCCA and *S100P* ^+^ *SPP1* ^-^ iCCA, with the gene-derived substance of *SPP1* being OPN. Immunohistochemical staining of a tissue microarray comprising 201 iCCAs revealed that OPN was positive in 92.0% (82/88) of small duct type iCCA patients and 3.4% (2/59) of large duct type iCCA patients (24). Yoshizawa T et al. further validated the utility of OPN in distinguishing iCCA subtypes using whole-section staining, demonstrating that OPN was positive in 100.0% (32/32) of small duct type iCCA patients and 12.2% (5/41) of large duct type iCCA patients, with all positive cases in the latter group being weakly positive (27). OPN is localized in the cytoplasm of tumor cells. The viewpoints on the significance of OPN in the biological behavior of iCCA are inconsistent. Terashi T et al. reported that low expression of OPN correlated with enhanced tumor invasiveness and poor prognosis (89), but Zheng Y et al. found that elevated OPN expression was associated with shorter overall survival and higher recurrence rates of iCCA by facilitating the growth and metastasis of iCCA (90). Laohaviroj M et al. noted that OPN expression in the tumor stroma was positively correlated with the malignant biological behavior of iCCA (91). These discrepancies may be attributed to differences in the subtypes of iCCA included in these studies.

TUBB3 is a microtubule protein that shares similarities with Nestin, which is typically expressed in neuron-derived cells. It is expressed in various types of tumors, including lung cancer, ovarian cancer, and esophageal cancer (92). For iCCA, two studies indicated that TUBB3 had potential value in differentiating the histological subtypes. Zen Y et al. found that TUBB3 was expressed in 50.0% (14/28) of small duct type iCCA patients, while the percentage of positive cases was conspicuously lower in the large duct type cases (15.0%, 6/40) (93). Another study conducted by Akita M et al. reported that the percentage of positive cases of TUBB3 in small duct type iCCA was 64.5% (20/31), compared to 31.6% (6/19) in the large duct type patients (22). TUBB3 staining is located in the cytoplasm of tumor cells. To date, no studies have focused on the prognostic significance of TUBB3 in iCCA.

Fibrinogen is primarily synthesized in the liver and is converted into fibrin through thrombin. During this process, fibrinogen releases FGB. FGB is recognized as an inflammation marker (94) and possesses similar functions to CRP. A study by Rhee H et al. found that FGB could serve as a valuable marker for small duct type iCCA, with a percentage of positive cases of 50.0% (10/20) in the small duct type iCCAs and 17.6% (18/102) in the large duct type iCCAs (19). Another study by Chung T et al. also indicated that the overall percentages of positive cases of FGB and CRP were significantly higher in small duct type iCCA (68). FGB is expressed in the cytoplasm of tumor cells, and its prognostic significance in iCCA remains unclear.

**Figure 2:**
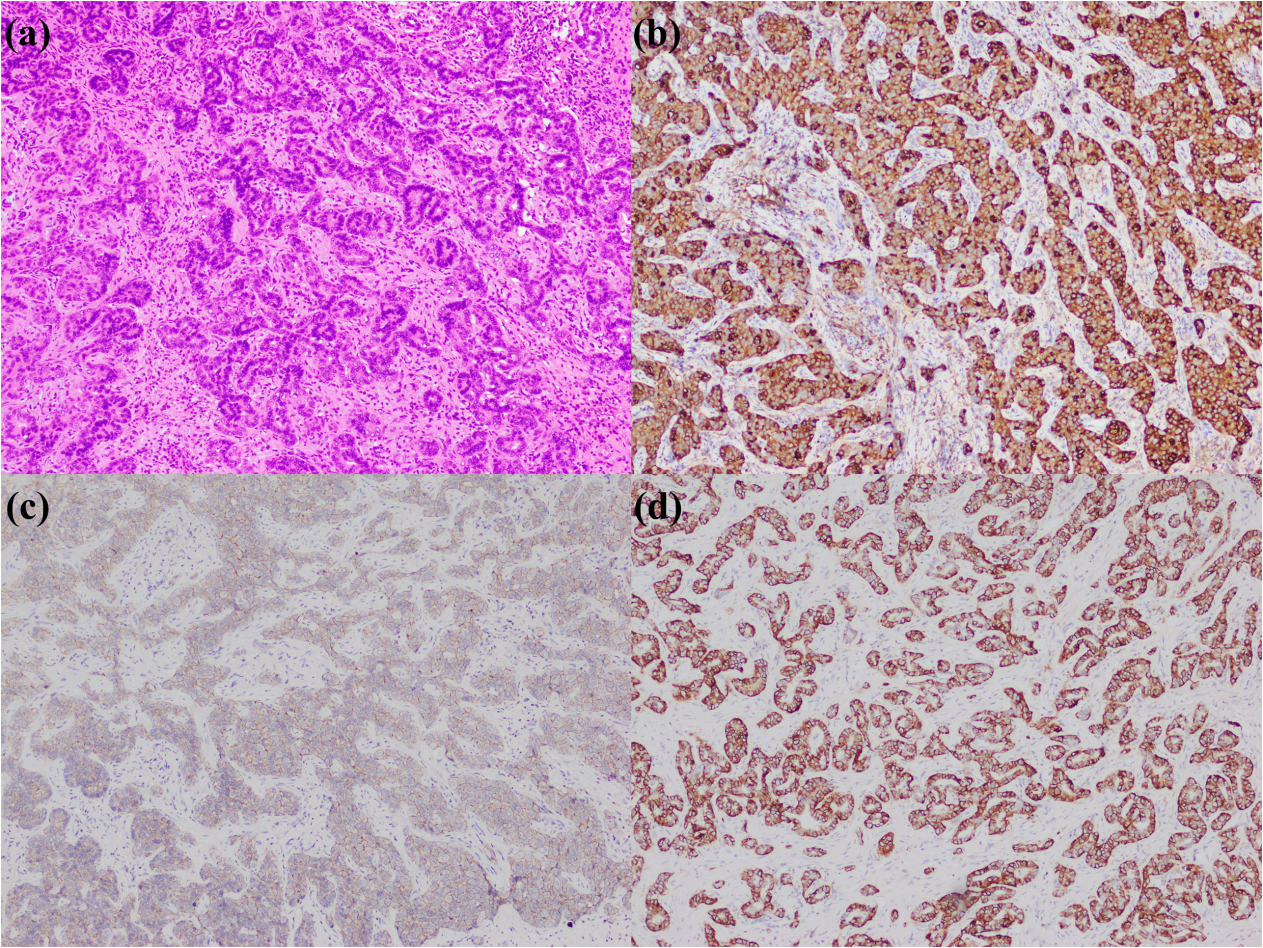
Small duct type iCCA. **(a)** histological morphology; **(b)** CRP positive; **(c)** N-cadherin positive; **(d)** CD56 positive (magnification: 100×).

In summary, the expression of immunohistochemical markers in small duct type iCCA is primarily observed in the cytoplasm of tumor cells and shows organ specificity, particularly for CRP and N-cadherin (**Figure 2**). This enables the application of these markers to extend from subtype diagnosis to differential diagnosis. Furthermore, the liver specifically synthesizes albumin, so albumin *in situ* hybridization has been increasingly performed for the diagnosis and differential diagnosis of liver tumors in recent years. Numerous studies showed that the percentage of positive cases of albumin *in situ* hybridization in small duct type iCCA is significantly higher than in large duct type iCCA, and both are higher than in metastatic adenocarcinoma. Consequently, albumin *in situ* hybridization can serve as a novel method for the histological subtype diagnosis and differential diagnosis of iCCA, supplementing immunohistochemistry (29, 52, 70, 95–98).

### 2.3 Cholangiolocarcinoma

**Figure 3:**
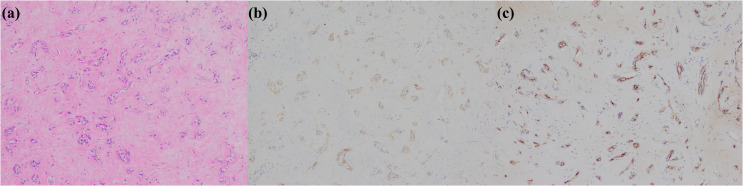
Cholangiolocarcinoma. **(a)** histological morphology; **(b)** CD56 positive; **(c)** MUC1 positive (magnification: 100×).

Cholangiolocarcinoma is composed by a well-differentiated ductular or cord-like structure resembling reactive bile ductules. The term “cholangiolocarcinoma” was first put forward in 1959 (99), and this entity was initially classified as a subtype of combined hepatocellular-cholangiocarcinoma (100). Nevertheless, multiple studies demonstrated that its morphological, immunohistochemical, and molecular characteristics are more akin to the small duct type iCCA (101–103). Therefore, in the 5th edition of the WHO Classification, cholangiolocarcinoma is reclassified as a distinct subtype of small duct type iCCA (2). To date, no specific immunohistochemical markers have been identified for cholangiolocarcinoma. A few studies with small sample sizes indicated that cholangiolocarcinoma frequently expressed CD56, with a percentage of positive cases ranging from 40.0% to 100.0% (20, 101, 102, 104, 105), while some researchers argued that the percentage of positive cases of CD56 in cholangiolocarcinoma did not remarkably differ from that in classical small duct type iCCA (101). The luminal expression pattern of MUC1 was another useful marker, although it was proved not to be specific (**Figure 3**) (16, 80, 101, 106, 107), similarly, the luminal expression of the L-selectin ligand MECA-79 mirrored MUC1 patterns and provided diagnostic utility (107). Nestin was another immunohistochemical marker that could assist in the diagnosis (85). One study even suggested that Nestin could serve as a differential marker between cholangiolocarcinoma and small duct type iCCA (80), although this view has not yet been widely accepted. Additionally, a recent study indicated that cholangiolocarcinoma had molecular alterations highly comparable to those of small duct type iCCA, and they found that cholangiolocarcinoma was notably characterized by mismatch repair-deficient (dMMR), leading to the loss of expression of MLH1, PMS2, MSH2, and MSH6, which could assist in differentiation (108).

### 2.4 iCCA with DPM pattern

**Figure 4:**
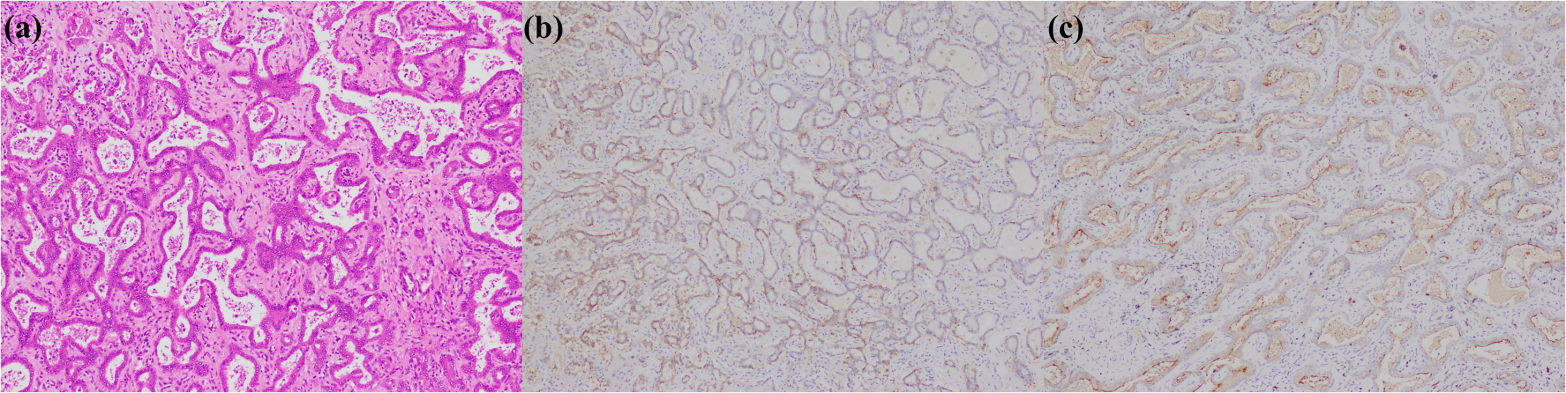
iCCA with DPM pattern. **(a)** histological morphology; **(b)** CD56 positive; **(c)** MUC1 positive (magnification: 100×).

Nakanuma Y et al. first introduced the term “iCCA with DPM pattern” in 2012 and classified it as a distinct subtype of small duct type iCCA. Histopathologically, this entity is characterized by irregularly dilated neoplastic bile ducts, resembling DPMs, and composed of single-layered columnar or low cuboidal cells with minimal cytoplasm and small nuclei lacking mitosis (109). This entity possesses some features of the hepatic progenitor cells, including the expression of epithelial cell adhesion molecule (EpCAM), cytokeratin (CK) 19, and CD56 (109, 110), leading some researchers to speculate that iCCA with DPM pattern shares commonness with cholangiolocarcinoma, both probably originating from the canals of Hering and bile ductules (80, 111). Pathological diagnosis of iCCA with DPM pattern primarily relies on morphological characteristics. The expression of CD56 (with a reported 100.0% percentage of positive cases in some small sample size studies) (80, 112, 113) and the luminal expression pattern of MUC1 (80) were valuable but not specific (**Figure 4**). Additionally, two studies found that *AT-rich interactive domain 1A* (*ARID1A*) mutation was closely associated with iCCA with DPM pattern. The immunohistochemical staining revealed that the percentage of absence or abnormal expression of ARID1A ranged from 40.0% to 57.9% (111, 113). It is important to emphasize that *ARID1A* mutations are not specific to this subtype and also occur in other histological subtypes of iCCA and even extrahepatic cholangiocarcinoma (114). Therefore, *ARID1A* mutations cannot be considered a specific gene variation for iCCA with DPM pattern.

### 2.5 Other rare subtypes of iCCA

**Figure 5:**
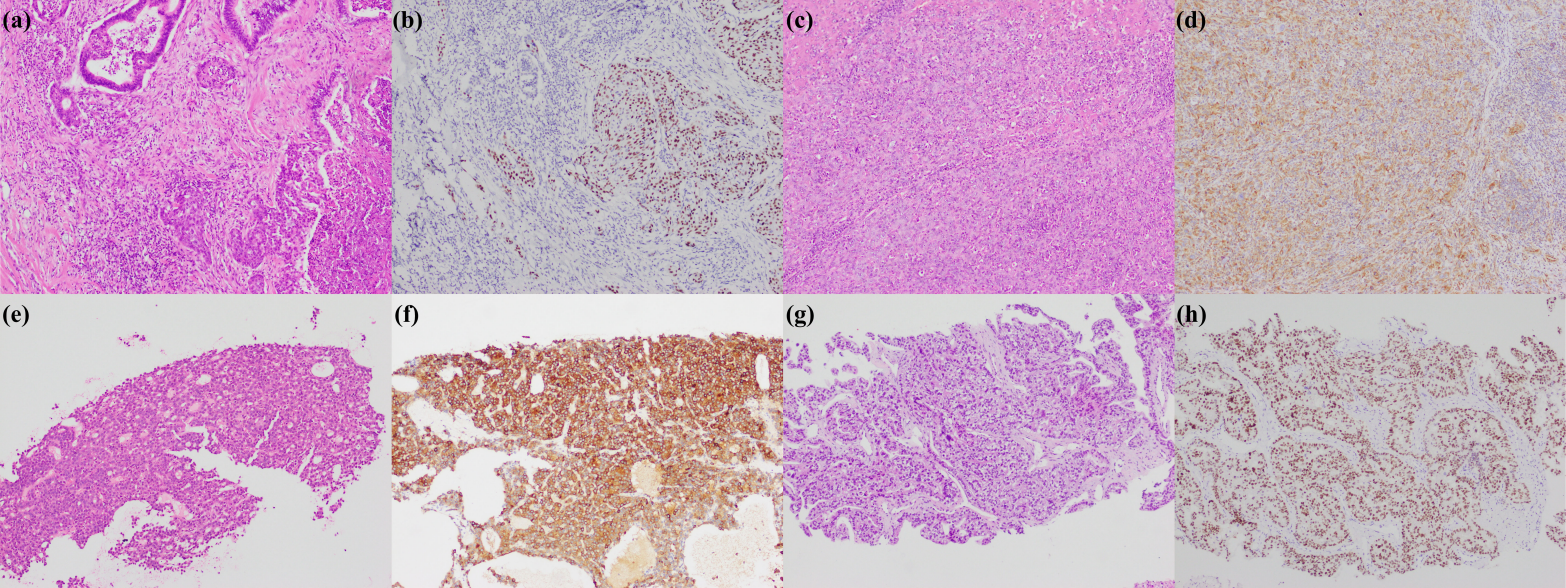
Rare subtypes of iCCA. **(a)** adenosquamous carcinoma; **(b)** p40 positive in squamous cell carcinoma region; **(c)** sarcomatous iCCA; **(d)** Vimentin positive in sarcomatous iCCA; **(e)** cholangioblastic iCCA; **(f)** α-inhibin positive in cholangioblastic iCCA; **(g)** enteroblastic iCCA; **(h)** SALL4 positive in enteroblastic iCCA (magnification: 100×).

The rare subtypes of iCCA were collected in the WHO Classification including adenosquamous and squamous cell carcinoma, mucinous carcinoma, signet ring cell carcinoma, clear cell carcinoma, mucoepidermoid carcinoma, lymphoepithelioma-like carcinoma, and sarcomatous iCCA (**Figure 5**) (2). Moreover, some new provisional subtypes of iCCA, such as cholangioblastic iCCA, enteroblastic iCCA, and acinar cell carcinoma, are also notable. There exist several useful immunohistochemical markers for identifying these iCCA entities. For instance, adenosquamous carcinoma or squamous cell carcinoma can be confirmed by p40 and p63. Sarcomatoid carcinoma can be identified by vimentin (115) and integrase interactor 1 (INI1), brahma-related gene 1 (BRG1), and other members of the switch defective/sucrose non-fermentable complex (23, 116). Cholangioblastic iCCA can be assessed using α-inhibin (117). Enteroblastic iCCA is characterized by the expression of spalt-like transcription factor 4 (SALL4) (118). Lymphoepithelial-like carcinoma requires confirmation by Epstein-Barr virus-encoded small ribonucleic acid (RNA) *in situ* hybridization. Immunohistochemical detection of latent membrane protein 1 (LMP1) is unreliable for ascertaining Epstein-Barr virus infection in iCCA (119).

### 2.6 Summary

The preceding sections summarize the current landscape of immunohistochemical markers used for histological subtyping of iCCA. Because of the different morphological interpretation criteria, antibody clones, positivity thresholds, and the preservation status of tumor tissues, the results across studies varied slightly, but few contradictory conclusions were reported. However, some established and emerging markers (e.g., MMP7, pCEA, CA19-9, CLDN18, CLDN18.2, Smad4/DPC4, EVI1, PPP1R1B, FGB), which were validated in a single study, still require more evidence through larger datasets. Based on WHO recommendations, reported discriminatory effectiveness, and our experience, we give our recommendations about immunohistochemical markers used in differentiating large duct type iCCA and small duct type iCCA (**Table 2**). Among the preferred markers, S100P, MUC5AC, CRP, and N-cadherin are also prioritized in the “Expert Consensus on Pathological Diagnosis of Intrahepatic Cholangiocarcinoma (2022 version)”. Additionally, OPN has been demonstrated with strong discriminatory performance and is supported by dual validation through molecular and immunohistochemical testing, making it a promising emerging marker for small duct type iCCA. Overall, small duct type iCCA markers demonstrate superior disease specificity compared to large duct type iCCA markers. The markers of large duct type iCCA are mainly used in the differential diagnosis with small duct type iCCA.

**Table 2:** Characterization of immunohistochemical markers of large duct type and small duct type iCCA.

Antibody	Corresponding subtype	Expression location	Percentage of positive cases	Established/emerging markers	Recommendation level
S100P	Large duct type iCCA	Nucleus and cytoplasm	Large duct type iCCA 68.5%~100.0% vs. Small duct type iCCA 0.0%~57.6%	Established marker	Preferred
MUC5AC	Large duct type iCCA	Cytoplasm	Large duct type iCCA 50.0%~96.7% vs. Small duct type iCCA 1.8%~25.0%	Established marker	Preferred
MUC6	Large duct type iCCA	Cytoplasm	Large duct type iCCA 13.7%~33.3% vs. Small duct type iCCA 3.8%~20.0%	Established marker	Optional
TFF1	Large duct type iCCA	Cytoplasm	Large duct type iCCA 54.9%~91.8% vs. Small duct type iCCA 12.0%~48.1%	Established marker	Optional
AGR2	Large duct type iCCA	Cytoplasm	Large duct type iCCA 58.9%~86.7% vs. Small duct type iCCA 19.5%~33.3%	Established marker	Optional
MMP7	Large duct type iCCA	Cytoplasm	Large duct type iCCA 91.2%	Established marker	Optional
pCEA	Large duct type iCCA	Cell membrane	Large duct type iCCA 66.7% vs. Small duct type iCCA 9.1%	Emerging marker	Optional
CA19-9	Large duct type iCCA	Cytoplasm	Large duct type iCCA 72.7% vs. Small duct type iCCA 8.4%	Emerging marker	Optional
CLDN18	Large duct type iCCA	Cell membrane	Large duct type iCCA 69.0% vs. Small duct type iCCA 12.7%	Emerging marker	Optional
CLDN18.2	Large duct type iCCA	Cell membrane	Large duct type iCCA 41.2% vs. Small duct type iCCA 10.0%	Emerging marker	Optional
Smad4/DPC4	Large duct type iCCA	Negative expression	Large duct type iCCA 33.3% vs. Small duct type iCCA 3.8%	Emerging marker	Optional
EVI1	Large duct type iCCA	Nucleus	Large duct type iCCA 78.6% vs. Small duct type iCCA 32.7%	Emerging marker	Optional
PPP1R1B	Large duct type iCCA	Cytoplasm	Large duct type iCCA 90.9% vs. Small duct type iCCA 4.0%	Emerging marker	Optional
CRP	Small duct type iCCA	Cytoplasm	Small duct type iCCA 65.0%~100.0% vs. Large duct type iCCA 5.3%~71.7%	Established marker	Preferred
N-cadherin	Small duct type iCCA	Cell membrane	Small duct type iCCA 55.0%~95.5% vs. Large duct type iCCA 5.6%~49.1%	Established marker	Preferred
CD56	Small duct type iCCA	Cell membrane and cytoplasm	Small duct type iCCA 16.2%~82.1% vs. Large duct type iCCA 0.0%~25.5%	Established marker	Optional
Nestin	Small duct type iCCA	Cytoplasm	Small duct type iCCA 40.9%~47.2% vs. Large duct type iCCA 3.3%~5.0%	Emerging marker	Optional
OPN	Small duct type iCCA	Cytoplasm	Small duct type iCCA 92.0%~100.0% vs. Large duct type iCCA 3.4%~12.2%	Emerging marker	Preferred
TUBB3	Small duct type iCCA	Cytoplasm	Small duct type iCCA 50.0%~64.5% vs. Large duct type iCCA 15.0%~31.6%	Emerging marker	Optional
FGB	Small duct type iCCA	Cytoplasm	Small duct type iCCA 50.0% vs. Large duct type iCCA 17.6%	Emerging marker	Optional

Established/emerging markers depend on whether they are recommended by the *WHO Classification of Tumours-Digestive System Tumours*.

The recommendation level is determined by the WHO recommendations, supported by robust sensitivity and specificity from multiple studies and our experience.

In some cases, co-expression of markers of different subtypes may occur, which may be attributed to several reasons. First, careful assessment of localization and extent of expression is essential; for instance, CRP-positive small glands in large duct type iCCA may represent residual peribiliary glands; although S100P is not uncommonly expressed in small duct type iCCA, the staining is usually weak, focal, and cytoplasmic. The published studies often employed semi-quantitative scoring systems based on both staining intensity and distribution. According to our experience and the methodologies reported in the literature, at least 10% of aggregated tumor cells exhibiting moderate-to-strong staining is considered meaningful, and the localization of staining should be accurate. Second, iCCAs originating at the junction between septal and area bile ducts may exhibit overlapping immunophenotypic and morphological features of both subtypes. In such cases, classification as hybrid/mixed subtypes is recommended. Pathologists should interpret immunohistochemical findings in the context of histological morphology and clinical data. When definitive subtyping is challenging, detailed documentation of expression patterns of markers—including distribution, intensity, and localization—should be included in the pathology report.

Currently, there is still a lack of immunohistochemical markers with high sensitivity and specificity for cholangiolocarcinoma and iCCA with DPM pattern. As for the former, there is morphological overlap with typical small duct type iCCA. It is recommended to apply a panel reflecting stem cell features in cases of well-differentiated iCCA with prominent sclerotic stroma to assist in the diagnosis of cholangiolocarcinoma. As for the latter, its morphological features are generally distinctive, and misdiagnosis is uncommon. Recent studies have proposed a subtype of cholangiocarcinoma associated with biliary adenofibroma, termed tubulocystic carcinoma of bile ducts (120). According to the histopathological images and molecular data presented in the literature, this entity exhibits a high similarity with iCCA with DPM pattern and may represent different terms of the same disease. This opinion has gained recognition among some researchers (121).

The subtyping of iCCA has gained increasing acceptance and application in China. This trend is not only influenced by the WHO recommendations, but also supported by extensive studies demonstrating that different iCCA subtypes have different genetic profiles, particularly in their drug-related molecular signatures, thereby generating significant clinical demand. At present, the identification of these genetic alterations mainly relies on molecular pathology techniques such as polymerase chain reaction (PCR), Sanger sequencing, fluorescence *in situ* hybridization (FISH), and NGS. Due to the high costs and technical complexity of these methods, several studies have explored immunohistochemistry for screening and achieved satisfactory sensitivity and specificity. This will be reviewed in the next part.

## 3 Targeted therapy and immunotherapy of iCCA

Following the latest guidelines and consensus, multiple drug-related targets were identified for iCCA, which can be utilized for primary/subsequent-line therapies of unresectable or metastatic biliary tract cancer (8, 122–126). These targets include *FGFR2* rearrangements, *IDH1* mutations, human epidermal growth factor receptor (*HER*) 2 overexpression/amplification, *v-raf murine sarcoma viral oncogene homologue B1* (*BRAF*) *V600E* mutation, *neurotrophin receptor kinase* (*NTRK*) fusions, *rearranged during transfection* (*RET*) fusions, *KRAS G12C* mutation, *mesenchymal-epithelial transition* (*MET*) amplification, *anaplastic lymphoma kinase* (*ALK*) fusions, *ROS proto-oncogene 1, receptor tyrosine kinase* (*ROS1*) fusions, phosphatase and tensin homolog (PTEN) deficiency, *neuregulin 1* (*NRG1*) fusions, pathogenic *breast cancer susceptibility gene* (*BRCA*) variation, microsatellite instability-high (MSI-H) or dMMR, high tumor mutational burden (TMB-H), and PD-L1 high expression. There is currently a paucity of immunohistochemistry-related studies on *KRAS G12C* mutation and pathogenic *BRCA* variation. Moreover, TMB-H, derived from NGS, cannot be assessed via immunohistochemistry, and thus are not discussed herein.

### 3.1 *FGFR2* rearrangements

**Figure 6:**
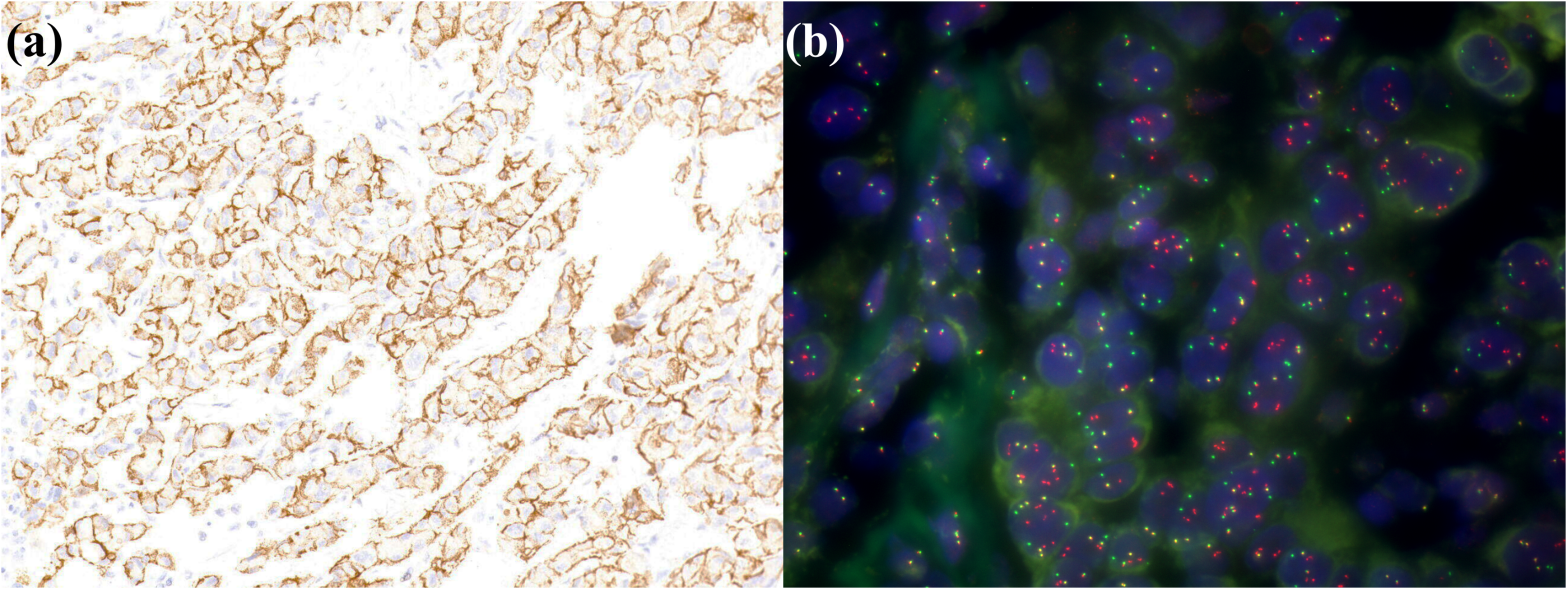
A small duct type iCCA patient with *FGFR2* rearrangements. **(a)** FGFR2 is positively expressed in tumor cells; **(b)** FISH demonstrates *FGFR2* breakage.

The *FGFR* family comprises four tyrosine kinase receptors—*FGFR1*, *FGFR2*, *FGFR3*, and *FGFR4*. Upon binding to specific growth factors, these receptors undergo dimerization, thereby activating downstream signalling pathways that regulate key processes such as cell proliferation, survival, and angiogenesis (127). *FGFR2* rearrangements mainly result in gene fusions and the incidence of *FGFR2* rearrangements in iCCA is approximately 10% to 15%, predominantly occurring in small duct type iCCA patients (3, 128, 129). Futibatinib, Pemigatinib, and Erdafitinib were validated effective for patients with biliary tract cancer with these molecular alterations (4, 130, 131) and were recommended by National Comprehensive Cancer Network as subsequent-line treatment regimen for unresectable and metastatic disease. The standardized methods for detecting *FGFR2* rearrangements include FISH and NGS (132), with RNA-NGS being particularly advantageous for identifying its partner genes. Several studies explored immunohistochemistry as a screening tool for *FGFR2* alterations in biliary tract cancer. Uson Junior PLS et al. initially used two FGFR2 antibodies (clone: FPR2-D and clone 98706) to stain 99 patients of biliary tract cancer. The first antibody identified 20 tumors with positive staining, while the second identified 10 tumors. The following verification using FISH or NGS confirmed that 14 of these tumors carried *FGFR2* alterations (13 fusions and 1 mutation). The immunohistochemical method demonstrated high accuracy (91.9% and 78.7%) and specificity (97.7% and 82.9%), along with moderate sensitivity (57.1% and 53.9%) (133). Additionally, Sasaki M et al. found that the percentage of positive cases of FGFR2 (clone: D4L2V) was significantly higher in small duct type iCCAs (25.7%, 9/35) than large duct type iCCAs (3.3%, 1/30) and hepatocellular carcinomas (0.0%, 0/35). They also noted that the 5’/3’ (E5/E18) imbalance in *FGFR2* (E5/E18 ratio > 2) was frequently observed in FGFR2-positive small duct type iCCAs, but less so in FGFR2-negative tumors (134). However, Zou Y et al. denied the screening value of the clone D4L2V of FGFR2. They analysed 167 iCCAs and found that 80 (47.9%) cases were positive for FGFR2, and 87 cases (52.1%) were negative. However, the immunohistochemical results showed inconsistency test for both FISH (κ = 0.048) and NGS (κ = 0.125) (135). Consistent with the findings of Zou Y et al., Cao Z et al. conducted an NGS analysis to identify *FGFR2* rearrangements in 9 out of 76 patients of iCCA. However, there was a relatively low consistency (κ = 0.464) between the results of NGS and immunohistochemistry of FGFR2 (clone: D4L2V) (136). These findings suggest that FGFR2 immunohistochemistry can serve as an available screening method for *FGFR2* rearrangements, but the clone of the antibody needs to be strictly selected. Regarding the prognostic significance, Uson Junior PLS et al. reported no statistically significant differences in overall survival, progression-free survival, and time to tumor recurrence between FGFR2-positive and FGFR2-negative patients (clone: FPR2-D and clone 98706) (133). However, another study involving 92 iCCA patients indicated that those expressing FGFR2 (clone: D4L2V) had worse recurrence-free survival (137). The discrepancies in these results may be attributed to different clones, highlighting the need for the development of more sensitive and specific FGFR2 antibodies (**Figure 6**).

### 3.2 *IDH1/2* mutations

The *IDH* family consists of three isoforms (*IDH1*, *IDH2*, and *IDH3*) that facilitate metabolic exchange and electron transfer between mitochondria and the cytosol. *IDH1/2* serve as the primary source of reduced nicotinamide adenine dinucleotide phosphate in most tissues. As a critical reducing agent, reduced nicotinamide adenine dinucleotide phosphate regulates antioxidant defense systems by neutralizing reactive oxygen species. Compromised *IDH1/2* activity in cancer cells may disrupt these detoxification processes, thereby inducing genomic instability through accumulated deoxyribonucleic acid lesions (138). *IDH* mutations, particularly *IDH1* mutations in iCCA patients, can benefit from Ivosidenib (recommended by National Comprehensive Cancer Network as subsequent-line treatment regimen for unresectable and metastatic biliary tract cancer) (5, 139). A small number of patients with iCCA also present with *IDH2* mutations. *IDH* mutations frequently occurr in small duct type iCCA patients. The common mutated sites for *IDH1* are at codon 132, and for *IDH2*, at codon 172. The incidence of *IDH1/2* mutations in iCCA is approximately 15% to 20% (140–143). Detection of *IDH* mutations can be accomplished through Sanger sequencing or NGS. The selection of suitable antibodies is of utmost importance because of the multiplicity of *IDH-mutated* sites. For instance, in a study employing the IDH1 antibody with clone OTI24A2, it was found that 92.9% (105/113) of iCCAs showed positive expression, failing to screen for related gene mutations (144). So far, two studies yielded encouraging results. In one study involving 95 iCCAs, *IDH1 R132* mutations were observed in 19 cases (20.0%), encompassing 5 cases of *IDH1 R132L*, 11 cases of *IDH1 R132C*, and 3 cases of *IDH1 R132G*. *IDH2 R172* mutations were confirmed in 2 cases (2.1%), including 1 case of *IDH2 R172M* and 1 case of *IDH2 R172K*. All *IDH1 R132L* mutation cases exhibited positive staining of IDH (clone: MsMab-2), while other *IDH1 R132* mutations, *IDH2* mutations, and wild-type samples were negative (145). In another study, Ma B et al. conducted sequencing of 130 patients with iCCA and revealed that 21 patients (16.1%) carried *IDH1/2* mutations, including 6 cases of *IDH1 R132C*, 5 cases of *IDH1 R132G*, 4 cases of *IDH1 R132H*, 2 cases of *IDH1 R132L*, 2 cases of *IDH2 R172K*, and 2 cases of *IDH2 R172W*. Positive expression of IDH (clone: MsMab-1) was detected in 14 patients (10.8%) of iCCA, which was specific for *IDH1 R132G*, *IDH1 R132H*, and *IDH2 R172W*. The sensitivity and specificity of this antibody for detecting the above three types of *IDH1/2* mutations were 81.8% (9/11) and 95.8% (114/119) (κ = 0.691) (21). The above two studies used different clones of IDH antibodies, and their combination may further enhance the detection efficacy. Unfortunately, these two studies did not show the prognostic significance of the expression of IDH. Moreover, the most prevalent *IDH1 R132C* mutation in iCCA (143) cannot be accurately detected by these antibodies. Further research is still necessary as the existing IDH antibodies are incapable of covering all types of *IDH* mutations.

### 3.3 *HER2* overexpression/amplification

**Figure 7:**
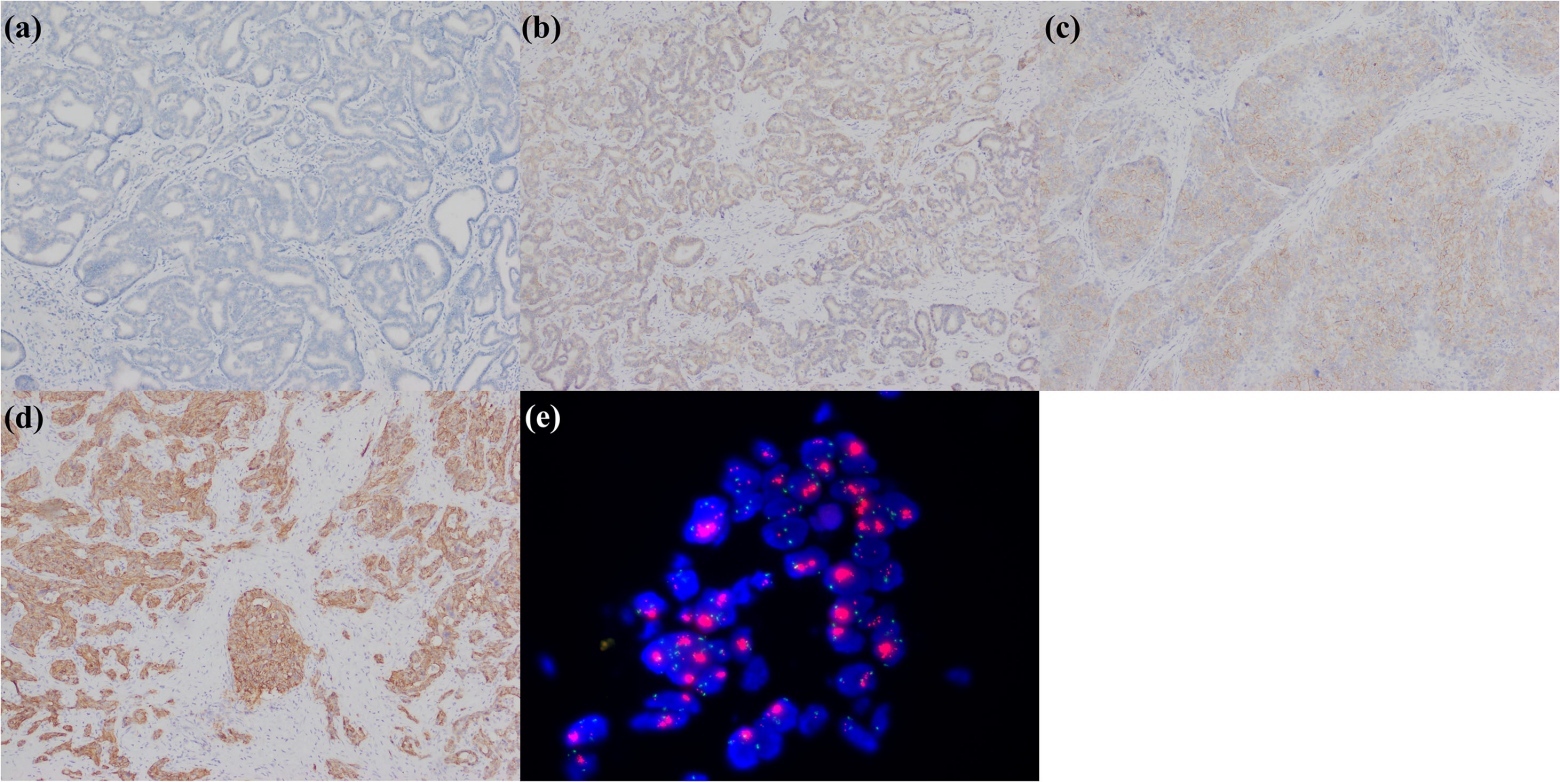
iCCA with heterogeneous HER2 status. **(a)** HER2 0; **(b)** HER2 1+; **(c)** HER2 2+; **(d)** HER2 3+; **(e)** FISH demonstrates *HER2* amplification.

The *HER*/*erythroblastic leukemia viral oncogene homolog* (*ERBB*) family proteins are type I transmembrane growth factor receptors that function to activate intracellular signaling pathways in response to extracellular signals. The members of *HER* family of kinases were shown to form both homodimers and heterodimers with each other, resulting in the activation of crucial cell signaling pathways, including the *phosphatidylinositol 3 kinase* (*PI3K*)/*protein kinase B* (*AKT*) pathway. *HER3* interacts directly with *PI3K*, and *HER2* indirectly activates this pathway through its interaction with *HER3*. The overexpression/amplification of *HER2* is implicated in multiple cancers (146). *HER2* detection can be achieved through immunohistochemistry, FISH, and NGS. HER2 immunohistochemistry has been widely employed in various cancer types such as breast cancer, gastrointestinal adenocarcinoma, and bladder cancer, and their standardized approaches are highly thorough. The commonly employed clone of HER2 includes 4B5, HercepTest, CB11, and so on. For biliary tract cancer, the high-grade evidence of approved HER2-targeted drugs is based on four clinical studies. The first one is the MyPathway study, where Pertuzumab combined with Trastuzumab was performed to treat metastatic biliary tract cancer patients with immunohistochemistry 3^+^ HER2, *HER2* amplification indicated by FISH or chromogenic *in situ* hybridization (*HER2*:*CEP17* ratio > 2.0, or *HER2* copy number > 6.0), or *HER2* amplification detected by NGS (*HER2* copy number gain) (147). The second is the SGNTUC-019 study, in which Tucatinib and Trastuzumab were used to treat metastatic biliary tract cancer patients with the same inclusion criteria as the MyPathway study (148). The third is Trastuzumab Deruxtecan for the treatment of advanced solid tumor patients with immunohistochemistry 3^+^ HER2 (149). The fourth study is HERIZON-BTC-01 which validated that Zanidatamab could improve the prognosis of the unresectable, locally advanced or metastatic biliary tract cancer patients with HER2 2^+^ and 3^+^, especially for the latter population (150). Regarding the percentage of positive cases of HER2 expression in iCCA, a meta-analysis including 40 studies showed that when the moderate/strong positive expression of HER2 was defined as positive, the percentage of positive cases of HER2 expression in iCCA was 4.8% (151). Another study incorporating 110 iCCAs suggested that when evaluated based on the guideline from the College of American Pathologists, American Society for Clinical Pathology, and the American Society of Clinical Oncology for gastroesophageal adenocarcinoma (152), the detection percentage of 2^+^ and 3^+^ HER2 expression in iCCA was 3.7% (153). One more study including 27 cases of iCCA indicated that when HER2 scoring was performed under the 2018 version of the American Society of Clinical Oncology/College of American Pathologists Clinical Practice Guideline (154), the detection percentage of 2^+^ and 3^+^ HER2 expression in iCCAs was also 3.7% (155). In terms of prognosis, a meta-analysis including 31 studies revealed that the overall survival of biliary tract cancer patients with HER2 overexpression was poorer, but when specified to the patients of iCCA, the conclusion still need further verification (156). Currently, there is no evidence for an independent HER2 immunohistochemical interpretation standard for iCCA, mainly referring to the methods of gastrointestinal adenocarcinoma and breast cancer. Considering that clear clinical evidence has been obtained through these methods, it is feasible from an empirical perspective (**Figure 7**). Additionally, although the majority of *HER2* alterations are gene overexpression or amplification, activating missense mutations have also been proven to be an important subset of *HER2* alterations, which cannot be detected by immunohistochemistry.

### 3.4 *BRAF V600E* mutation

**Figure 8:**
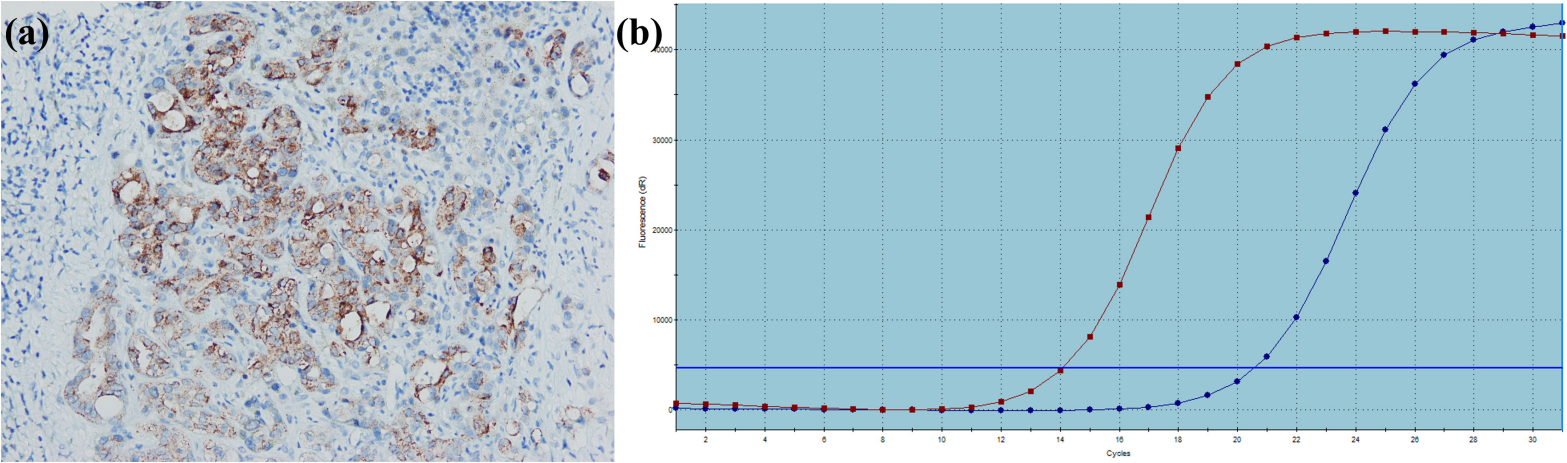
A large duct type iCCA patient with *BRAF V600E* mutation. **(a)** BRAF is positively expressed in tumor cells; **(b)** PCR demonstrates *BRAF V600E* mutation.

BRAF is a serine/threonine kinase and a critical node in the canonical mitogen-activated protein kinase cascade. Activated BRAF phosphorylates mitogen activated protein kinase 1/2, which, once activated, subsequently phosphorylate extracellular signal-regulated kinase 1/2. The activated extracellular signal-regulated kinase 1/2 then phosphorylate downstream effectors that regulate cell proliferation, survival, and various other cellular processes. When *BRAF* undergoes mutation, it leads to constitutive activation of this pathway, thereby promoting tumorigenesis (157). In 2020, the results of Dabrafenib combined with Trametinib for the treatment of *BRAF V600E* mutant biliary tract cancer publised, reporting an approximately 50% objective response rate, and the vast majority of the enrolled patients were iCCA (39/43, 90.7%) (158). Therefore, it was approved by National Comprehensive Cancer Network for the treatment of advanced biliary tract cancer patients as a subsequent-line regimen. The mutation rate of *BRAF V600E* in iCCA is approximately 1.1% to 3.0% (159–161). PCR, Sanger sequencing, and NGS are currently the standard approaches for detecting this mutation. The immunohistochemical detection of *BRAF V600E* mutation yielded excellent results in non-small cell lung cancer, thyroid cancer, colorectal cancer, and melanoma (162–165), which spurred an exploration in biliary tract cancer. Sasaki M et al. utilized its specific antibody VE1 staining in 17 patients with small duct type iCCA and found no positive expression, and no *BRAF V600E* mutation was identified through PCR detection (166). Goeppert B et al. also employed VE1 to detect 159 patients of iCCA, 149 patients of extrahepatic cholangiocarcinoma, and 69 patients of carcinoma of the gallbladder, and discovered 5 patients with positive expression, all of whom were iCCAs (5/159, 3.1%). Further PCR for *BRAF V600E* mutation was highly consistent with the immunohistochemical results (167). Regarding prognosis, Goeppert B et al. asserted that *BRAF V600E* mutation was not correlated with the prognosis of biliary tract cancer (167), while Wu S et al. suggested that BRAF V600E (clone: RM8) was positively expressed in 10.0% of iCCA patients (11/110), and the prognosis of BRAF V600E positive iCCA patients was better, but they did not verify the consistency between positive expression and molecular mutation (38). To sum up, immunohistochemical detection using the BRAF V600E antibody holds potential as an effective means for the selection of related targeted therapies for patients with iCCA (**Figure 8**).

### 3.5 *NTRK* fusions

The TRK receptor family include TRKA, TRKB, and TRKC (encoded by *NTRK1*, *NTRK2*, and *NTRK3*, respectively), all of which share a highly homologous sequences with conserved structural organization. TRK receptors bind neurotrophin family ligands, a group of highly homologous dimeric growth factors involved in the development and maintenance of the nervous system (168). *NTRK* fusions were initially discovered in colon cancer and subsequently identified in various tumor types (169). Entrectinib (170), Larotrectinib (171), and Repotrectinib (172) were recommended by the National Comprehensive Cancer Network for the primary/subsequent-line therapies of biliary tract cancer with *NTRK* fusions. Considering that the occurrence rate of *NTRK* fusions in biliary tract cancer is less than 1%, rational testing is required to screen for relevant patients. The recommended testing method is NGS, especially RNA-NGS, and the related immunohistochemical antibody pan-TRK (clone: EPR17341) is also applied in practice. In the initial studies, it was thought that pan-TRK immunohistochemistry could effectively recognize solid tumors with *NTRK* fusions and could present three staining patterns: nuclear staining, cytoplasmic and nuclear membrane staining, or cytoplasmic and cytoplasmic membrane staining (173, 174). However, with the increased exploitation, it was found that its sensitivity to *NTRK3* fusions was limited, and there were a considerable number of false-positive cases (175). For biliary tract cancer, a study involving 351 Caucasian patients revealed that no pan-TRK-positive staining was detected (clone: EPR17341) (176). In another study encompassing 85 Asian patients (42 patients of iCCA), pan-TRK positive expression was observed in 26 patients (13 patients with iCCA) with 25 weakly positive (clone: EPR17341) (177). The above two studies imply that pan-TRK expression may vary by ethnicity. Zhang D et al. conducted pan-TRK testing on 69 patients of iCCA and 110 patients with hepatocellular carcinoma. The former group showed no positive expression and 12 patients of hepatocellular carcinoma showed weak expression of tumor cells in the cytoplasm, but no *NTRK* fusions were detected through molecular testing (clone: EPR17341) (178). Demols A et al. tested 140 samples of biliary tract cancer and indicated that 17 cases showed positive expression, among which 11 were iCCA. However, only 1 case of perihilar cholangiocarcinoma was found to have *NTRK* fusions through NGS testing, and its immunohistochemical staining was weakly positive (clone: EPR17341) (179). The above results suggest that pan-TRK immunohistochemical staining can currently only function as a rough screening approach. Given its notable false-positive rate, any intensity and pattern of staining should not be overlooked, and further NGS testing for positive expression cases is necessary.

### 3.6 *RET* fusions

*RET* encodes for a transmembrane receptor tyrosine kinase with proto-oncogene properties. *RET* binds to the ligand-co-receptor complex of glial cell line-derived neurotrophic factor family ligands. *RET* signaling is vital for renal morphogenesis, neural and neuroendocrine tissue development, and spermatogonial stem cell maintenance. *RET* fusions are a type of somatic mutation leading to the formation of distinct RET oncoproteins. The Phase I/II ARROW clinical trial gauged the effectiveness of Pralsetinib for patients having *RET* fusions-positive solid tumors. Out of the 28 assessable patients, three had biliary tract cancer, and two obtained a verified response (180). The Phase I/II LIBRETTO-001 study appraised the efficacy of Selpercatinib in *RET* fusions-positive solid tumors and included one patient with biliary tract cancer (181). Both Pralsetinib and Selpercatinib were integrated into the National Comprehensive Cancer Network guidelines as primary/subsequent-line treatment alternatives for advanced biliary tract cancer featuring *RET* fusions (8). The “Expert consensus on the diagnosis and treatment of *RET* fusion non-small cell lung cancer in China” (182) and the “Chinese expert consensus on the diagnosis and treatment of advanced *RET* fusion-positive non-small cell lung cancer (2023 edition)” (183) suggested detecting *RET* fusions through NGS, PCR, FISH, and immunohistochemistry. They recommended that NGS and PCR were favored due to greater sensitivity and specificity. As of now, no studies have evaluated RET immunohistochemistry in iCCA. Immunohistochemistry screening for *RET* fusions showed passable sensitivity and specificity in the studies of non-small cell lung cancer (clone: 6E4C4; clone 3F8; EPR2871) (184–187). Another study based on 41,869 tumors indicated that the specificity of RET immunohistochemistry (clone: EPR2871) was 82.0% (73/89). The detection sensitivity varied by fusion partner: *KIF5B* had the highest sensitivity (100.0%, 31/31), followed by *CCDC6* (88.9%, 16/18) and *NCOA4* (50.0%, 6/12) (188). These results highlight the potential of immunohistochemistry in the detection of *RET*-fusions for iCCA patients.

### 3.7 *MET* amplification

**Figure 9:**
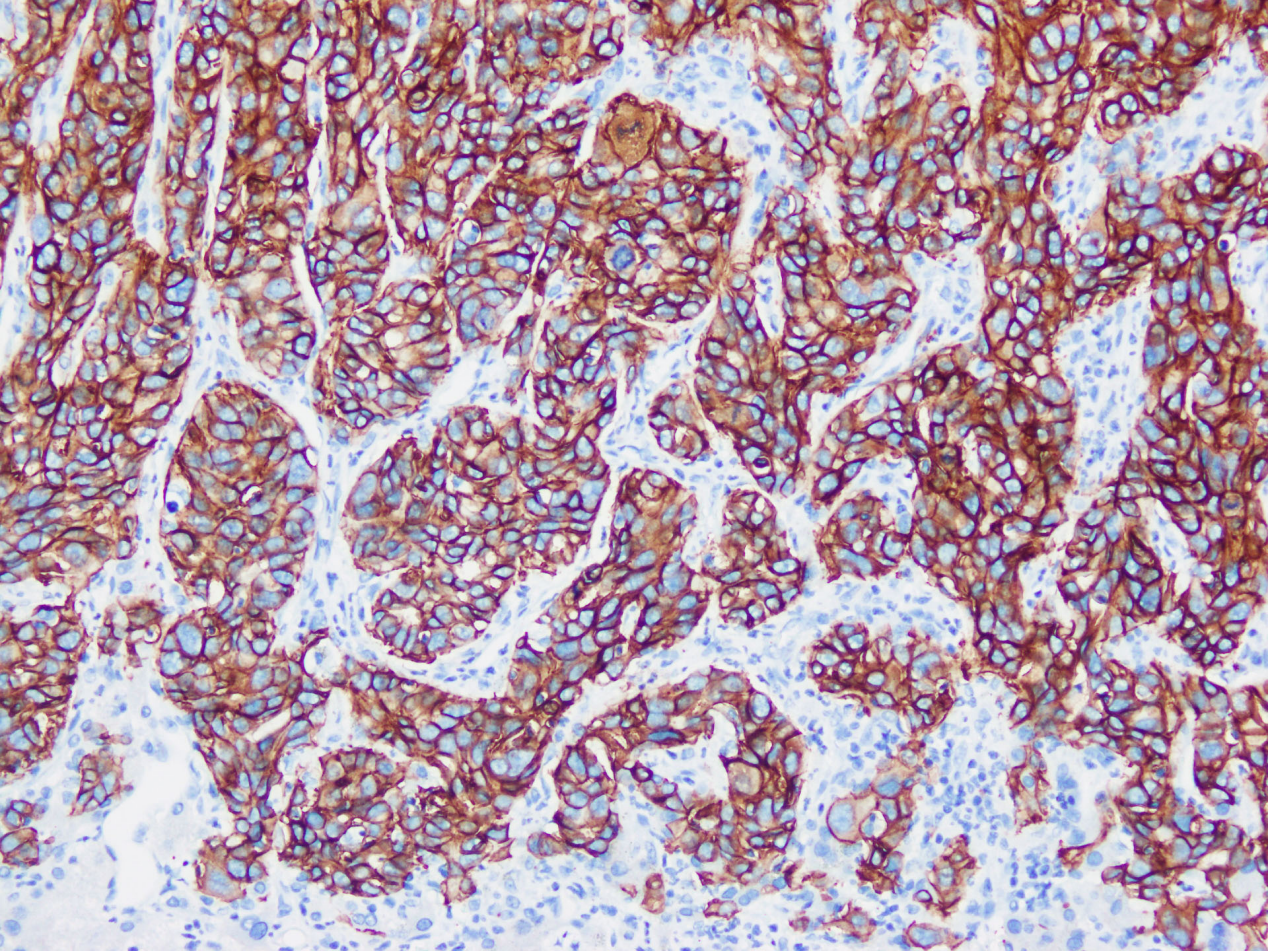
A small duct type iCCA patient with *MET* amplification harbored by NGS exhibits strong MET expression in tumor cells.

The *MET* proto-oncogene encodes the tyrosine kinase receptor of the hepatocyte growth factor and modulates critical biological processes including embryonic development, tissue repair, hepatic regeneration, vascular remodeling, and immune regulation. In cancer, dysregulated MET signaling drives tumor progression through its ability to enhance invasive potential, stimulate blood vessel formation, and facilitate metastatic dissemination (189). *MET* amplifications, mutations, and fusions are drivers of oncogenesis. Although no clinical trials have affirmatively demonstrated that iCCA with *MET* amplification can gain benefits from *MET* inhibitors, many preclinical studies and case reports pointed out its potential (190–200). *MET* variations are not scarce in iCCA. An NGS analysis of 28 iCCAs showed that the rate of *MET* variations was 7.1% (201). Another study on 349 advanced iCCAs indicated that the rate of *MET* amplification was 4.6% (202). One more study covering 6,130 iCCAs suggested that the proportion of *MET* amplification was 2.0% (142). Existing studies explored the MET expression in iCCA and its connection with tumor biological behavior, prognosis, and molecular alterations. Regarding tumor biological behavior, two studies performed MET immunohistochemical staining in iCCAs and documented that the expression was stronger in well-differentiated tumors than in poorly differentiated ones (203, 204). However, Joo HH et al. held that MET expression was directly related to tumor invasiveness in biliary tract cancer (205). Heo MH et al. believed MET expression was not associated with the clinicopathological characteristics of biliary tract cancer (206). In terms of prognosis, three studies discovered that the percentage of high expression of MET in iCCA ranged from 6.8% to 51.5% and was significantly related to the poor prognoses (207–209). Regarding the consistency of MET expression and *MET* amplification, a study involving 133 iCCAs revealed that 21 cases (15.8%), 41 cases (30.8%), and 71 cases (53.4%) had high-frequency *MET* amplification, low-frequency *MET* amplification, and normal *MET*, respectively. Among the high-frequency *MET* amplification cases, 3 tumors had cluster amplification, and they all showed strong MET expression. Strong staining was not observed in tumors with other genetic alterations. The proportion of MET positive expression in high-frequency *MET* amplification patients was higher than that in low-frequency *MET* amplification and normal *MET* patients (52.4% vs. 22.0% vs. 8.5%) (210). Another exploration of 27 iCCAs showed that 4 had *MET* amplification, 6 showed MET positive expression to varying degrees, and 1 had *MET* amplification when MET staining was negative (155). Overall, immunohistochemical detection of MET expression can provide a preliminary reference for patients with *MET* amplification to some extent. Recently, the “Chinese expert consensus on clinical practice of MET detection in non-small cell lung cancer” was issued, recommending FISH and NGS as the standardized methods to validate *MET* amplification and proposing a scoring approach for MET immunohistochemical evaluation integrating expression range and intensity, and preferentially using the SP44 clone MET antibody (211), which can be referred to (**Figure 9**).

### 3.8 *ALK* fusions

*ALK* encodes a highly conserved receptor tyrosine kinase in the insulin receptor superfamily and leads to the activation of downstream signaling pathways critical for cellular proliferation, survival, and differentiation. In several cancers, *ALK* fusions have been identified as key oncogenic drivers (212). *ALK* inhibitors have made significant progress in non-small cell lung cancer and can be accurately identified through immunohistochemical detection (213). Even though *ALK*-related molecular alterations are seldom seen in iCCA (214–217), preclinical studies and case reports verified the existence of *ALK* fusions in this tumor entity and the possible benefit from *ALK* inhibitors (197, 218–220). The reported gene fusions in iCCA included *EML4*::*ALK* and *STRN*::*ALK* (197, 218, 219, 221). Among them, two papers provided ALK immunohistochemical results, both of which were positive (clone: D5F3) (219, 221). Another study included 80 iCCAs for ALK immunohistochemical staining (clone: ZAL4) with 55 cases showing positive expression, but only 5 cases showed strong expression. Further FISH detection revealed that one of them had *ALK* rearrangement, but no detailed fusion gene data were provided (209). Based on these studies, immunohistochemistry for screening patients with iCCA with *ALK* fusions has potential value, but it needs to be clarified by FISH or NGS.

### 3.9 *ROS1* fusions

*ROS1* is located at 6q22 and encodes a receptor tyrosine kinase in the subclass of the insulin receptor family. The function of ROS1 in human physiology remains unclear. Genetic fusions in *ROS1* result in constitutive activation of the tyrosine kinase, serving as a driver for tumor proliferation (222). *ROS1* fusions are rare in iCCA (223). Reported fusion genes in iCCA include *TMEM106B*::*ROS1*, *FIG*::*ROS1*, and *RDX*::*ROS1* (224–227). Preclinical studies and case reports suggested the targeted therapeutic significance of *ROS1* fusions (197, 226). The expression of ROS1 in iCCA and its connection with molecular alterations were documented in three literatures. The immunohistochemical staining of ROS1 (clone: D4D6) in 194 iCCAs by Lee KH et al. disclosed positive expression in 72 cases. Positive expression of ROS1 was associated with well-differentiated tumors and great disease-free survival, but no *ROS1* fusion was detected through FISH (228). The immunohistochemical staining of ROS1 in 85 cases of iCCA by Chiang NJ et al. indicated positive expression in 57 cases (67.1%) and strong positive expression in 9 cases (10.6%). However, they thought positive expression of ROS1 was positively correlated with the unfavorable prognosis, and no *ROS1* fusion cases were detected by FISH either (209). The immunohistochemical staining of ROS1 (clone: D4D6) in 198 cases of iCCA by Lim SM et al. manifested positive expression in 38 cases and identified that 3 cases had ROS1 rearrangement, but one of them was ROS1 negative (229). Given this, both the recognition capability of ROS1 immunohistochemical staining and its correlation with prognosis remain unclear, and its utilization should be prudent.

### 3.10 PTEN deficiency

*PTEN* regulates many cellular processes, including proliferation, survival, energy metabolism, cellular architecture, and motility. The tumor-suppressor activity of *PTEN* depends largely on its lipid phosphatase activity, which inhibits *PI3K*/*AKT* activation (230). For the genesis of malignant tumors, *PTEN* stands as one of the most common genes. The absence of PTEN expression is not a rare occurrence in iCCA. Lee D et al. demonstrated that 67 cases showed the loss of PTEN expression among 101 iCCAs, and the prognosis of such cases was unfavorable (clone: 138G6) (231). Jiang TY et al. identified that 30 patients had loss of expression of PTEN (clone: 138G6) among 98 advanced iCCAs (232). For patients with advanced iCCA with the loss of PTEN expression, they further probed into the therapeutic value of Bortezomib. Among the 130 included patients, 38 patients (29.2%) were affirmed to have the absent or low expression of PTEN (clone: 138G6). The objective response rate and disease control rate of the 13 patients who eventually received Bortezomib treatment were 23.1% and 53.9%, respectively. The median progression-free survival was 3.6 months, and the median overall survival was 9.6 months, which surpassed the prognosis of patients with high PTEN expression. The three patients with notable tumor shrinkage were all with complete loss of PTEN expression (233). Currently, the study on PTEN and iCCA remains limited, and immunohistochemical testing of PTEN has yet to be widely used in routine clinicopathological practice for iCCA. Based on our experience, PTEN frequently exhibits weakly positive staining with ambiguous localization in tumors, leading some pathologists to interpret such cases as negative. This risks significant false-negative interpretation. We propose that strict criteria should be established to distinguish patients with PTEN deficiency, and further validation should be conducted in larger cohorts of iCCA.

### 3.11 *NRG1* fusions

*NRG1* is critical for cell differentiation and organogenesis (234). *NRG1* fusions act as driver gene in solid tumors. The connection between *NRG1* and *HER3* boosts heterodimerization with other kinases of the *HER*/*ERBB* family, thus strengthening downstream signaling pathways and tumorigenesis. As a result, focusing on the *ERBB* pathway becomes a hopeful therapeutic approach for cancers with *NRG1* fusions (235). Zenocutuzumab, an antibody-dependent cellular cytotoxicity-enhanced anti-*HER2* and *HER3* bispecific antibody, proved effective in treating solid tumors harboring *NRG1* fusions and received endorsement from the National Comprehensive Cancer Network in the treatment of pancreatic cancer (236). A phase II clinical trial evaluating its safety and efficacy in *NRG1* fusions-positive advanced solid tumors indicated a partial response in one of three cholangiocarcinoma patients (237). Although *NRG1* fusions are seldom seen in iCCA (238), there has been case reports noting successful treatments in related patients (239). In 2024, Chinese scholars issued the “Expert consensus on the diagnosis and treatment of *NRG1/2* fusion solid tumors” proposing RNA-NGS and pERBB3 immunohistochemistry as efficient methods for detecting *NRG* fusions (240). Notably, pERBB3 immunohistochemistry showed high sensitivity and specificity in lung adenocarcinoma (clone: Tyr1289) (241, 242). Comparable studies in iCCA are currently absent.

### 3.12 MSI-H/dMMR

**Figure 10:**
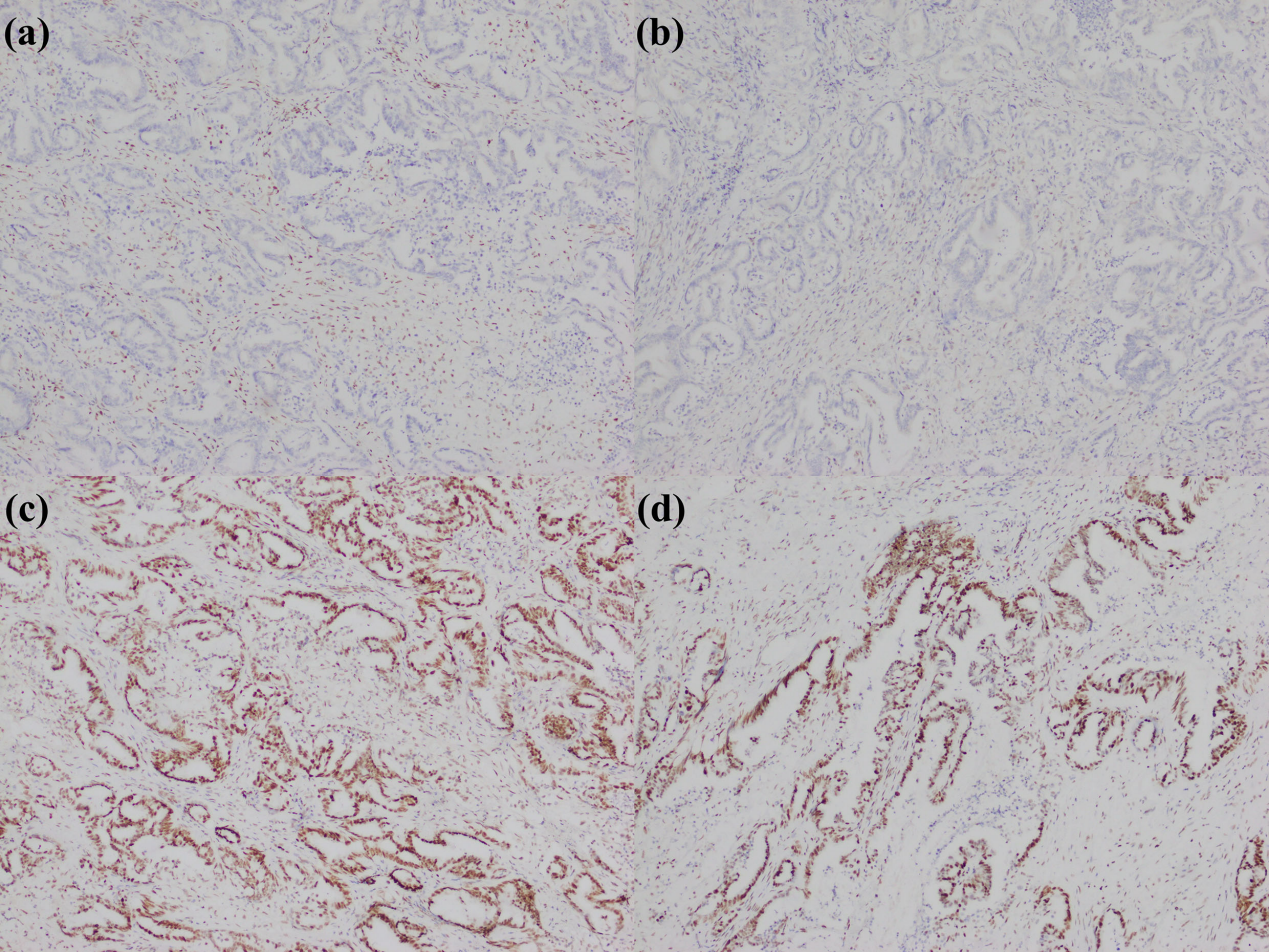
A large duct type iCCA patient with dMMR. **(a)** loss of MLH1 expression; **(b)** loss of PMS2 expression; **(c)** MSH2 expression; **(d)** MSH6 expression.

The DNA MMR system is responsible for the identification, excision, and repair of base-pair mismatches or indel loops in the genome. Loss of expression of any of the MMR proteins involved in this process will disrupt genomic stability, leading to MSI and is associated with a higher risk for tumorgenesis (243). The success and approval of Pembrolizumab (244) and Dostarlimab (245) in the treatment of solid tumors with MSI-H or dMMR prompted many academic societies to formulate guidelines for the detection of dMMR tumors (246–248). These guidelines unanimously propose three methods: PCR for MSI, immunohistochemistry for MMR (MLH1, MSH2, MSH6, and PMS2), and NGS. Among them, immunohistochemistry is regarded as the most convenient method with the complete absence of expression of any one or more of the four proteins in tumor cells indicating dMMR (**Figure 10**). The prevalence of dMMR in biliary tract cancers is approximated to be 2% to 3% (249). Nevertheless, large-scale investigations specifically focusing on the incidence of dMMR in iCCA are scarce. A study encompassing 584 iCCAs reported an MSI-H rate of 6.0% (n=35), with patients demonstrating enhanced prognosis after immunotherapy (250). An NGS-based study discovered an MSI-H rate of 1.3% (75/5,885) in iCCAs (142). From the aspect of immunohistochemistry, Winkelmann R et al. did not find dMMR among 35 iCCA patients (251). Yu J et al. identified dMMR in 2.7% (n=2) of 73 iCCAs (252). A study of 71 iCCAs determined that 9.9% (n=7) had at least one MMR protein lacking, which correlated with a histology of mucinous adenocarcinoma and a poor prognosis (253). Khuntikeo N et al. discovered that 27.3% (n=21) of 77 liver fluke-related iCCAs were with dMMR, suggesting a favorable prognosis for these patients (254). This detection rate of dMMR was not in line with the MSI-H rate in another group of liver fluke-related iCCA patients (255), but the prognostic implications were consistent with the findings of Cloyd JM et al. (256). The actual incidence of dMMR in iCCA remains to be fully clarified. Existing evidence substantiated the clinical advantage of immunotherapy for dMMR-positive patients (250, 257–260). Hence, routine detection for MLH1, MSH2, MSH6, and PMS2 in iCCA patients is highly significant. Although the three detection methods demonstrate substantial concordance, studies in other tumor types have revealed a discordance rate of <15% between MMR and MSI testing results (261–263). The main reasons for the inconsistency include: functional redundancy among MMR proteins (e.g., MSH3 can compensate for isolated MSH6 loss, preserving partial MMR activity and MSS status), missense mutations and false-positive of MLH1, suboptimal immunohistochemical staining quality, and the low tumor cellularity (264). Given these potential differences, the simultaneous use of immunohistochemistry and PCR, or even NGS, might offer more reliable data (265).

### 3.13 PD-L1 high expression

**Figure 11:**
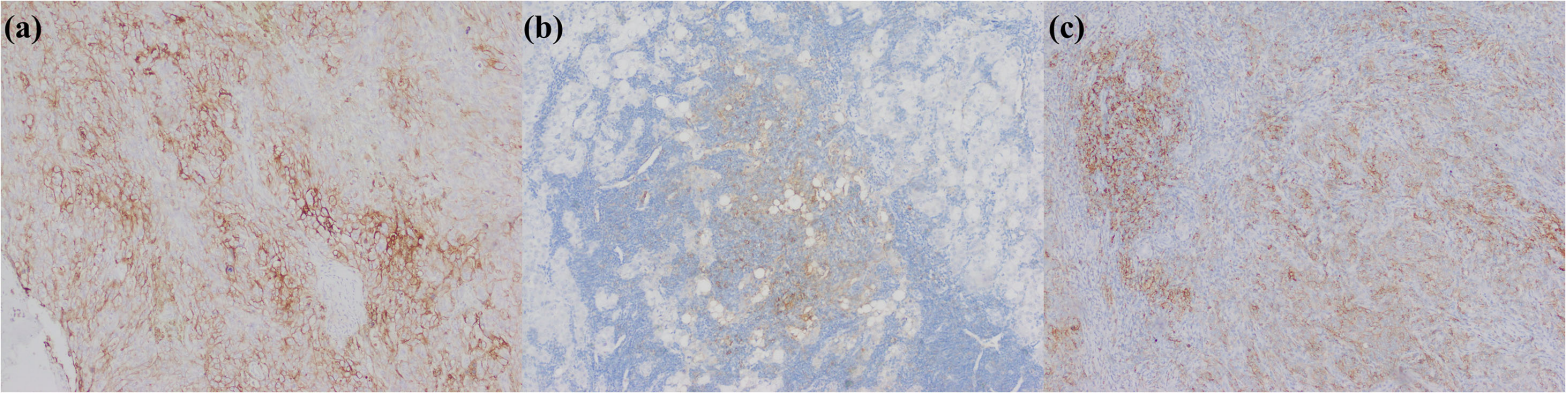
iCCA with heterogeneous PD-L1 expression. **(a)** PD-L1 is positively expressed in tumor cells; **(b)** PD-L1 is positively expressed in immunocytes; **(c)** PD-L1 is positively expressed in tumor cells and immunocytes.

PD-L1 is notably expressed across tumor-associated cell populations, including neoplastic cells, stromal components, and myeloid lineages (**Figure 11**). Through dual ligand interactions with programmed death-1 receptors on T cells and CD80 co-stimulatory molecules on antigen-presenting cells, PD-L1 inhibits T cell proliferation, cytokine production, and cytolytic activity driving T cells toward a dysfunctional exhausted state (266). Although PD-L1 inhibitor Durvalumab has been approved for primary preferred treatment of biliary tract cancer, the connection between elevated PD-L1 expression and the therapeutic outcome of immunotherapy for iCCA remains uncertain but this relationship warrants attention. A study manifested that PD-L1 expression in biliary tract cancer could serve as a potential predictor for the therapeutic efficacy of Pembrolizumab. The objective response rate and disease control rate were higher in the high PD-L1 expression group [tumor-positive score (TPS) ≥ 50%] (267). In a single-arm phase II clinical trial of Camrelizumab combined with Gemcitabine and Oxaliplatin for the treatment of patients with advanced biliary tract cancer, the objective response rate of patients with PD-L1 TPS ≥ 1% was 80.0%, outperforming that of 53.8% of patients with PD-L1 TPS < 1% (268). A meta-analysis encompassing 30 studies investigated the predictive value of PD-L1 expression for the response to immunotherapy in biliary tract cancer and suggested that there was no significant distinction in the objective response rate and disease control rate between PD-L1^+^ and PD-L1^-^ patients. Nevertheless, the progression-free survival and overall survival were enhanced in the former group. Within the included studies, the determination of PD-L1 positivity was mainly based on TPS ≥ 1% or combined positive score (CPS) ≥ 1 (269). Another meta-analysis incorporating 10 studies revealed that high PD-L1 expression was positively correlated with poorer overall survival and recurrence-free survival in iCCA (270). Hence, it is rationally supposed that although the prognosis of iCCA with high PD-L1 expression is unfavorable, immunotherapy may improve the prognosis of this subgroup. There were some proposed interpretation standards and validated clones for PD-L1 immunohistochemistry in recent years (271–273), which are used to assess PD-L1 evaluation in iCCA.

### 3.14 Summary

The screening of drug-related molecular targets by immunohistochemistry in iCCA remains nascent. Among the aforementioned immunohistochemical markers, HER2, BRAF, and MMR proteins have demonstrated great concordance with molecular testing in iCCA. Diffuse and strong HER2 membranous staining, diffuse BRAF cytoplasmic staining, and absence of MMR proteins expression are indicative of potential survival benefits from corresponding targeted therapies and immunotherapies.

Furthermore, the correlation between PD-L1 expression levels and the clinical efficacy of immune checkpoint inhibitors in iCCA has not yet been definitively established. This may be attributed to several factors, including heterogeneous PD-L1 expression across tumor cells, variability in detection platforms and scoring criteria, as well as complex interactions with other molecules within the tumor microenvironment. It is therefore recommended that, when feasible, PD-L1 testing be performed on multiple paraffin blocks representing different regions from the same case, in order to obtain a more comprehensive profile and provide clinicians with enhanced insights.

For immunohistochemical markers of other molecular alterations, their current utility remains limited. There are still many conflicting reports for the same marker, which might be attributed to discrepancies in antibody clones (leading to variations in sensitivity and specificity), differences in histological subtypes of study populations, and variations in interpretation methodologies. It is recommended that definite staining with any intensity, localization, and distribution warrant further confirmation through molecular testing, and detailed information regarding expression should be documented. In cases which immunohistochemical findings are discordant with molecular testing, appropriate explanations should be provided to both patients and clinicians, with molecular testing serving as the optimal standard currently. In addition to the continuous optimization of existing antibody clones to more precisely correspond with the associated molecular alterations, for gene alternations lacking antibodies, such as *KRAS G12C* mutation, the development of effective immunohistochemical detections is meaningful. Moreover, standardizing interpretation methods and implementing rigorous quality control standards are essential to ensure consistency in microscopic assessments for pathologists.If immunohistochemistry can be effectively aligned with molecular testing in the future, it will fundamentally transform the economic costs, execution efficiency, and clinical trial enrollment criteria for selecting targeted therapies and immunotherapies in patients with iCCA. A comprehensive summary of drug-related immunohistochemical antibodies is provided in **Table 3**.

**Table 3:** Appropriate immunohistochemical markers for drug targets in iCCA.

Drug targets	Drugs	Approval status	Standardized methods	Antibodies	Appropriate clones	Expression location	Recommendation level for antibodies
*FGFR2* rearrangements	Futibatinib, Pemigatinib, Erdafitinib	Approved	NGS, FISH	FGFR2	FPR2-D, clone 98706	Cell membrane	Optional
*IDH1* mutations	Ivosidenib	Approved	NGS, Sanger sequencing	IDH	MsMab-2, MsMab-1	Cytoplasm and/or cell membrane, Cytoplasm and nucleus	Optional
*HER2* overexpression/amplification	Pertuzumab+Trastuzumab, Tucatinib+Trastuzumab, Trastuzumab Deruxtecan	Approved	NGS, FISH, Immunohistochemistry	HER2	4B5, HercepTest, CB11, etc.	Cell membrane	Preferred
*BRAF V600E* mutation	Dabrafenib+Trametinib	Approved	NGS, PCR, Sanger sequencing, Immunohistochemistry	BRAF	VE1	Cytoplasm	Preferred
*NTRK* fusions	Entrectinib, Larotrectinib, Repotrectinib	Approved	NGS	pan-TRK	EPR17341	Nucleus/cytoplasm and nuclear membrane/cytoplasm and cell membrane	Optional
*RET* fusions	Pralsetinib	Approved	NGS, PCR	RET	6E4C4; clone 3F8; EPR2871	NOS	Optional
*MET* amplification	Tivantinib, Crizotinib, Capmatinib, Savolitinib, Tepotinib	Unapproved	NGS, FISH	MET	SP44	Cell membrane	Optional
*ALK* fusions	Alectinib, Crizotinib, Ensartinib, Ceritinib, Crizotinib	Unapproved	NGS, FISH, Immunohistochemistry	ALK	D5F3, ZAL4	Cytoplasm	Optional
*ROS1* fusions	Crizotinib	Unapproved	NGS, FISH	ROS1	D4D6	Cytoplasm	Optional
PTEN deficiency	Bortezomib	Unapproved	Immunohistochemistry	PTEN	138G6	Negative expression	Optional
*NRG1* fusions	Zenocutuzumab	Unapproved	NGS, FISH	pERBB3	Tyr1289	Cell membrane and/or cytoplasm	Optional
dMMR/MSI-H	Pembrolizumab, Dostarlimab	Approved	NGS, PCR, Immunohistochemistry	MLH1, PMS2, MSH2, MSH6	Multiple panels of clones	Negative expression	Preferred
PD-L1 high expression	Durvalumab	Approved	Immunohistochemistry	PD-L1	22C3, 28-8, SP263, etc.	Cell membrane (tumor cell and/or immunocyte)	Optional

Recommendation level for antibodies is mainly based on the concordance between immunohistochemical and molecular assays.

## 4 Conclusions

Immunohistochemistry, serving as a practical and cost-effective method, is indispensable for the contemporary pathological management of iCCA. We summarize distinct immunohistochemical profiles that are significantly related to the identification of histological subtyping. However, based on current findings, there is still a lack of highly specific immunohistochemical markers for large duct type iCCA, cholangiolocarcinoma, and iCCA with DPM pattern. This warrants further exploration in future studies. Since the histological subtyping of iCCA is closely correlated with the frequency of molecular alterations, accurate histological subtyping can provide more valuable clues and facilitate the screening of drug-related molecular targets. While molecular pathological techniques remain essential for definitively detecting of many genetic alterations, the immunohistochemical markers summarized herein offer an alternative approach for drug-target identification, especially in resource-limited settings. However, many immunohistochemical antibodies targeting drug-associated targets continue to demonstrate only poor correlations with molecular findings. It is necessary to focus on developing highly specific antibodies for emerging therapeutic targets to assist in the treatment selection of iCCA. Pathologists and clinicians should be aware that histological morphology and clinical information are essential prerequisites for any pathological diagnosis when utilizing immunohistochemistry for diagnosis. The positive range, localization, intensity, and appropriate controls of immunohistochemical staining are all indispensable considerations to ensure accurate interpretation.

## Glossary

AGR: Anterior gradient protein
AKT: Protein kinase B
ALK: Anaplastic lymphoma kinase
ARID1A: AT-rich interactive domain 1A
BRAF: V-raf murine sarcoma viral oncogene homologue B1
BRCA: Breast cancer susceptibility gene
BRG1: Brahma-related gene 1
CA19-9: Carbohydrate antigen 19-9
CDH2: Cadherin 2
CK: Cytokeratin
CLDN: Claudin
CPS: Combined positive score
CRP: C-reactive protein
dMMR: MMR-deficient
DPM: Ductal plate malformation
EpCAM: Epithelial cell adhesion molecule
ERBB: Erythroblastic leukemia viral oncogene homolog
EVI1: Ecotropic virus integration site 1 protein homolog
FGB: Fibrinopeptide B
FGFR: Fibroblast growth factor receptor
FISH: Fluorescence *in situ* hybridization
HER: Human epidermal growth factor receptor
iCCA: Intrahepatic cholangiocarcinoma
IDH: Isocitrate dehydrogenase
INI1: Integrase interactor 1
KRAS: Kirsten ratsarcoma viral oncogene homolog
LMP1: Latent membrane protein 1
MET: Mesenchymal-epithelial transition
MMP7: Matrix metalloproteinase 7
MSI-H: Microsatellite instability-high
MUC: Mucin
NGS: Next-generation sequencing
NRG1: Neuregulin 1
NTRK: Neurotrophin receptor kinase
OPN: Osteopontin
pCEA: Polyclonal carcinoembryonic antigen
PCR: Polymerase chain reaction
PD-L1: Programmed cell death ligand 1
PI3K: Phosphatidylinositol 3 kinase
PPP1R1B: Protein phosphatase 1 regulatory inhibitor subunit 1B
PTEN: Phosphatase and tensin homolog
RET: Rearranged during transfection
RNA: Ribonucleic acid
ROS1: ROS proto-oncogene 1, receptor tyrosine kinase
S100P: S100 calcium-binding protein P
SALL4: Spalt-like transcription factor 4
Smad4/DPC4: SMAD family member 4
SPP1: Secreted phosphoprotein 1
TFF1: Trefoil factor 1
TMB-H: Tumor mutational burden
TPS: Tumor-positive score
TUBB3: Class III β-tubulin
WHO: World Health Organization
